# FOXD1 regulates cell division in clear cell renal cell carcinoma

**DOI:** 10.1186/s12885-021-07957-8

**Published:** 2021-03-24

**Authors:** Kyle H. Bond, Jennifer L. Fetting, Christine W. Lary, Ivette F. Emery, Leif Oxburgh

**Affiliations:** 1 The Rogosin Institute, 310 East 67th Street, New York, NY 10065 USA; 2 Graduate School of Biomedical Sciences and Engineering, University of Maine, 168 College Ave, Orono, 04469 ME USA; 3 Maine Medical Center Research Institute, 81 Research Drive, Scarborough, ME 04074 USA; 4 Current affiliation: ICON Plc, 2100 Pembrook Parkway, North Wales, 19446 PA USA

**Keywords:** Cell cycle, DNA damage, Forkhead, Kidney cancer

## Abstract

**Background:**

Forkhead transcription factors control cell growth in multiple cancer types. *Foxd1* is essential for kidney development and mitochondrial metabolism, but its significance in renal cell carcinoma (ccRCC) has not been reported.

**Methods:**

Transcriptome data from the TCGA database was used to correlate *FOXD1* expression with patient survival. *FOXD1* was knocked out in the 786-O cell line and known targets were analyzed. Reduced cell growth was observed and investigated in vitro using growth rate and Seahorse XF metabolic assays and in vivo using a xenograft model. Cell cycle characteristics were determined by flow cytometry and immunoblotting. Immunostaining for TUNEL and γH2AX was used to measure DNA damage. Association of the *FOXD1* pathway with cell cycle progression was investigated through correlation analysis using the TCGA database.

**Results:**

*FOXD1* expression level in ccRCC correlated inversely with patient survival. Knockout of *FOXD1* in 786-O cells altered expression of FOXD1 targets, particularly genes involved in metabolism (*MICU1*) and cell cycle progression. Investigation of metabolic state revealed significant alterations in mitochondrial metabolism and glycolysis, but no net change in energy production. In vitro growth rate assays showed a significant reduction in growth of 786-O^FOXD1null^. In vivo, xenografted 786-O^FOXD1null^ showed reduced capacity for tumor formation and reduced tumor size. Cell cycle analysis showed that 786-O^FOXD1null^ had an extended G2/M phase. Investigation of mitosis revealed a deficiency in phosphorylation of histone H3 in 786-O^FOXD1null^, and increased DNA damage. Genes correlate with *FOXD1* in the TCGA dataset associate with several aspects of mitosis, including histone H3 phosphorylation.

**Conclusions:**

We show that FOXD1 regulates the cell cycle in ccRCC cells by control of histone H3 phosphorylation, and that FOXD1 expression governs tumor formation and tumor growth. Transcriptome analysis supports this role for FOXD1 in ccRCC patient tumors and provides an explanation for the inverse correlation between tumor expression of *FOXD1* and patient survival. Our findings reveal an important role for FOXD1 in maintaining chromatin stability and promoting cell cycle progression and provide a new tool with which to study the biology of FOXD1 in ccRCC.

**Supplementary Information:**

The online version contains supplementary material available at 10.1186/s12885-021-07957-8.

## Background

Forkhead transcription factors play diverse roles in cancer cells [1]. Members of the FOXA, C, M, O, and P subfamilies are involved in cancer initiation, progression and drug resistance. Expression of several forkhead family members has been described in clear cell renal cell carcinoma (ccRCC), a highly angiogenic tumor originating from nephron epithelium in the cortex of the kidney [2, 3].

The forkhead transcription factor FOXD1 plays an essential role in kidney development [4], and because developmental programs are frequently misregulated in cancer, we were interested to know what role it plays in cancer of the adult kidney, specifically ccRCC which is the most common form. Studies in other types of cancer suggest that it may be an important determinant of tumor biology. FOXD1 is highly expressed in Hodgkin’s lymphoma and in breast cancer, where it suppresses p27 expression, thereby promoting cell proliferation [5]. In non-small cell lung cancer, *FOXD1* expression correlates with poor survival and increases proliferation [6]. In glioma, FOXD1 expression correlates with tumor grade and influences proliferation and migration of cells [7]. FOXD1 expression is also downregulated in chemoresistant ovarian cancers [8].

In this study, we defined an inverse correlation between probability of survival in ccRCC patients and expression of *FOXD1* in their tumors. A knockout was generated to investigate the biological function of *FOXD1* in ccRCC cells. Expression of the known transcriptional target *MICU1*, a regulator of mitochondrial bioenergetics, was increased. While a shift in balance between mitochondrial respiration and glycolysis was seen, overall energy production in null cells was unaltered. Inactivation of *FOXD1* caused a significant reduction in cellular proliferation both in vitro and in tumor xenograft assays, and studies of cells with synchronized cycling times revealed that the G2/M phase of the cell cycle was extended in mutant cells. Transcriptional pathway analysis in patient tumors supported this finding, and investigation of phosphorylation state of histone H3 revealed that it is almost completely abrogated in *FOXD1* null cells. We propose that *FOXD1* is required for histone H3 phosphorylation, which is an essential step in the transition through G2/M.

## Methods

### Experimental model and subject details

#### Cell lines

786-O (ATCC® CRL-1932™) cells and 786-O ^FOXD1null^ were maintained in RPMI 1640 with 1x GlutaMax, 25mM HEPES, 10% FBS, and 1x penicillin-streptomycin in 37 °C cell culture incubators at 5% CO_2_. Cells were passaged for expansion using TrypLE express. For immunostaining, cells were grown on gelatin-coated sterile coverslips. Mycoplasma testing was performed prior to all experimental testing.

#### In vivo animal studies

Animal care was in accordance with the National Research Council Guide for the Care and Use of Laboratory Animals, and all experiments were approved by the Institutional Animal Care and Use Committee of New York Blood Center, where the Rogosin Institute laboratory is located. All animal experiments followed the housing protocols of the New York Blood Center: Complete health surveillance testing is performed for each murine room quarterly. Interim testing is performed as necessary. Serology is performed by Charles River Laboratories. Parasitology is performed in-house. The mice are housed in ventilated microisolator cages with 1 cup of 1/8 in. Bed-o'cobs. Mice are on 5P76 rodent feed and acidified water. Full PPE including head and shoe covers, isolation gown, mask and gloves, are required before room entry. All procedures are done inside a hood. Once animals leave the room, they do not return. Cages and water bottles are changed weekly and other cage materials are changed every 2 weeks. 6 week old female NCr nude mice (CrTac:NCr-*Foxn1nu*) were purchased from Taconic and housed at the New York Blood Center animal facility. Animals were housed in groups of 3–4 per cage. To establish xenografts, 786-O cells and 786-O^FOXD1null^ were expanded, resuspended at 10 million cells per 200 μl PBS and injected subcutaneously in the right flanks of 6-week old NCr mice, 6 animals per cell type. 48 h post-injection, tumor volume was measured using electronic calipers (V = LxW^2^). Tumor establishment was determined 7 days from first measurement, as determined from pilot studies (Fig. S3). Tumor growth was monitored every 2–3 days for 60 days. At the end-point, animals were euthanized with isoflurane followed by cervical dislocation.

### Methods detail

#### TCGA data analysis

Level 3 RNA sequencing data from the Illumina HiSeq platform was downloaded from the TCGA data portal (www.cancergenome.nih.gov) for 20,532 genes and 528 unique samples. We used the RPKM (reads per kilobase mapped) as gene expression values and linked this data to the clinical patient data using the patient barcode. Kaplan-Meier analyses were performed by stratifying gene expression data for each gene at the median and tested using the log-rank statistic. The survdiff and survtest R functions were using for Kaplan-Meier analyses, and the coxph function was used for fitting a Cox Proportional Hazards model to each gene to obtain the hazard ratio.

#### Patient samples

One hundred and forty two de-identified formalin-fixed, paraffin-embedded ccRCC samples from resected tumors were obtained from the Maine Medical Center BioBank, which obtains surgical samples with the written informed consent of patients. This study was approved by the Maine Medical Center Institutional Review Board (IRB #4202X and IRB #4392).

#### Immunohistochemistry

Section immunostaining was conducted as previously described [9]. Primary antibody FOXD1 (1:100, LSBio LS-B6453) and biotinylated rabbit anti-goat (1:500, Vectorlabs BA-5000) were used for immunohistochemistry using Vectastain ABC Elite kit (Vectorlabs PK-7100), and the color reaction performed using DAB. FOXD1 immunofluorescence in embryonic sections was amplified using the TSA amplification kit per manufacturer’s instructions (Perkin Elmer NEL756001KT).

#### FIJI analysis and quantification of expression

To quantify molecular marker expression, immunostained tumor sections were imaged at five separate regions, and the percentage of stained cells in each image was calculated using FIJI and used to calculate FOXD1 staining confidence ratio. To assign FOXD1+ scores to individual tumors, the mean percentage of FOXD1+ cells from all five images was calculated.

#### Generation of the 786-O^FOXD1null^ line

786-O cells (ATCC: CL-188; 70,003,535) were the parental cells used to generate a pool of *FOXD1*-null cells. CRISPR/Cas9-mediated knockout cells were generated by the Synthego Corporation (Cambridge, MA, USA). In brief, guide RNAs were designed to create premature stop codons through frameshift mutations in the coding region via insertions and/or deletions (Indels) directly upstream of the start codon of *FOXD1*. The following sgRNA sequence was used: *5′ AUCGGACAUCUCAGUGCUCA-3′ [GGG]-PAM*. To generate these cells, ribonucleoproteins (RNPs) containing the Cas9 protein and synthetic sgRNA were electroporated into the cells. Editing efficiency was assessed upon recovery, 48 h post-electroporation. Genomic DNA was extracted, PCR-amplified, sequenced using Sanger sequencing, and analyzed for editing efficiency using Synthego Inference of CRISPR Edits (ICE) software (ice.synthego.com). Limiting dilution was performed to generate 84 clonal populations and screened for indels using ICE software as described above. Top scoring lines were analyzed for FOXD1 protein expression using immunocytochemistry described below.

#### qPCR analysis of predicted FOXD1 targets

RNA was extracted from 786-O and 786-O^FOXD1null^ cells at 70% confluency using Qiagen RNeasy Minikit (Qiagen Cat#74134) with DNase step included. cDNA was generated using BioRad iScript Synthesis kit (BioRad Cat#1708891). A screen of 31 reference genes was performed using BioRad SsoAdvanced Universal SYBR Green Supermix using the manufacturer’s protocol, and selection of appropriate reference genes was performed using BioRad CFX Maestro software Reference Gene Selector Tool. *RPLPPO*, *HBB*, and *B2M* were chosen for this application. qPCR primers of predicted FOXD1 target genes were assayed and fold changes were calculated using BioRad CFX Maestro software using parental 786-O as reference. All assays were performed on CFX384 or CFX96 Touch Real-Time PCR Detection Systems, in triplicates.

#### Immunocytochemistry analysis

Immunostaining for FOXD1 in cells is described in brief; cells were fixed in 4% PFA for 12 min at room temperature, permeabilized with 0.2% Triton X-100 for 10 min at room temperature and blocked with 10% chicken serum in PBS for 1 h at room temperature. Binding of FOXD1 primary antibody (1:100, LSBio Cat#LS-B6453), biotinylated chicken anti-goat (1:250, ImmunoReagents Cat#CkxGt-003-EBio), and Cy5-conjugated streptavidin (1:250, Jackson Immunoresearch Cat#016–170-084) were performed sequentially at room temperature, 1 h each step and counterstained with DAPI. Coverslips were mounted using ProLong Diamond (Invitrogen Cat#P36961) mounting medium. The staining protocol was modified for phosphoproteins γH2AX (1:400, EMD Millipore Cat#05–636) and pH3 (1:100, EMD Millipore Cat#06–579) as follows; cells were fixed in ice cold 100% methanol and blocked overnight with 10% goat serum and 0.1% BSA in TBS. Primary antibody incubation was performed overnight, followed by secondary antibody incubation in goat anti-mouse IgG1 Alexa Fluor 568 (Life Technologies Cat#A21124) and goat anti-rabbit Alexa Fluor 647 (Life Technologies Cat#A21245). Coverslips were counterstained with DAPI, phalloidin Oregon-green (Invitrogen Cat#07466), or EdU (Invitrogen Cat#C10337) as pertaining to experiment. Staining for mitochondria using TOMM20 (1:100, abcam Cat# ab186735) was performed using standard immunocytochemistry techniques. Coverslips were mounted in EverBrite Mounting Media (Biotium Cat#23001). All staining was repeated to confirm staining patterns.

#### In vitro growth rate analysis

Five thousand cells of 786-O parent or 786-O^FOXD1null^ were plated into each well of a 6-well plate. Every 24 h for 4 days, cells were trypsinized and counted in triplicates. After 4 days, cells were collected and counted every 48 h. This experiment was repeated 3 times.

#### DNA damage analysis (TUNEL)

786-O or 786-O^FOXD1null^ cells were plated in 8-well chamber slides and cultured for 24 h. Cells were then fixed in 4% paraformaldehyde, permeabilized with 2:1 ethanol:acetic acid, and DNA damage was detected using the ApopTag® Red In Situ Apoptosis Detection Kit (Millipore Cat#S7165). In brief, damaged DNA was tagged with digoxigenin-dNTP, then detected using a fluorescently conjugated anti-digoxigenin antibody. Cells were counterstained with DAPI. Extent of DNA damage was analyzed by counting the number of TUNEL positive foci per nucleus in approximately 300 cells in each group.

#### Metabolic analyses

Two thousand cells of 786-O and 786-O^FOXD1null^ were plated into Seahorse XF96 Cell Culture Microplates and assayed using XF Glycolytic Rate Assay (*N* = 2) (Agilent Cat#103592–100), XF Cell Mito Stress Test (*N* = 3) (Agilent Cat#103015–100), or XF ATP Real-Time rate assay (*N* = 2) (Agilent Cat#103020–100) following manufacturer procedures. Six technical replicates were analyzed for each biological replicate.

#### EdU pulse-chase analysis

786-O parent and 786-O^FOXD1null^ were treated (“pulse”) with 5 μM EdU for 6, 8, or 10 h. After treatment, cells were washed and media was replaced with standard cell culture media. Cells were cultured (“chased”) for an additional 0, 2, 4, or 6 h. At each time point, cells were fixed in 100% ice cold methanol and stored in TBS at 4 °C until all time points were collected. Following all collections, cells were stained using the ICC protocol above, and counterstained with DAPI. EdU was detected following the manufacturer’s protocol. Cells were imaged using a Leica Thunder Imager. Cells were analyzed for co-expression of EdU, γH2AX, and pH3 using the Leica LasX multi-channel analysis package.

#### Cell cycle analysis

786-O parent and 786-O^FOXD1null^ were synchronized using double thymidine block. In brief, cells were treated for 16 h with thymidine, washed, and cultured for 9 h. A second round of thymidine was added for an additional 16 h to synchronize cells into G1. Cells were washed and fresh media was added. Cells were collected at various time points and either fixed in 100% ice cold methanol or protein extracted using Laemmli Buffer. Following fixation, cells were stained overnight with DAPI at 4 °C before flow cytometry. Flow cytometry was conducted at New York Blood Center Flow Cytometry Core Facility. Cell cycle distribution was performed on FlowJo software. Entry into mitosis was determined by Western blot analysis staining for H3 (CST Cat#9706 L), pH3(Ser10) (CST Cat#9715), CDC2 (CST Cat# 28439S), pCDC2(Tyr15) (CST Cat#9111S), pCDC2 (Thr161) (CST Cat#9114S), and CyclinB1 (CST Cat#12231). Molecular weight and densitometry analysis were performed using BioRad Imagelab software and normalized to total protein loaded.

#### In silico FOXD1 molecular signaling pathway prediction analysis

Gene correlation values were ranked for correlation strength and analyzed using Gene Set Enrichment Analysis (GSEA), mapping pathway interactions using the Reactome database and further analyzed using Gene Ontology (GO) analysis [10]. Pathways and interactors were mapped using Cytoscape [11]. Genes involved in pathways grouped under “Cell Cycle” were summarized and marked for further investigation.

#### Quantification and statistical analysis

For in vivo experiments, 3–4 animals were used per group. Due to the low tumor retention rate after 2 weeks of 786-O^FOXD1null^ (12.5%), statistical comparisons between end-point tumor size at 60 days were not possible, and instead comparisons at 15 and 30 days were performed, using Student’s two tailed t-test. All in vitro experiments utilized the Student’s t-test for comparisons between 786-O and 786-O^FOXD1null^. Correlation analyses were conducted using either the Pearson or Spearman rank correlation as implemented with the R function cor.test, and chi-squared tests were conducted to test the association between dichotomized variables.

## Results

### *FOXD1* expression in ccRCC correlates with poor patient survival

**Fig. 1:**
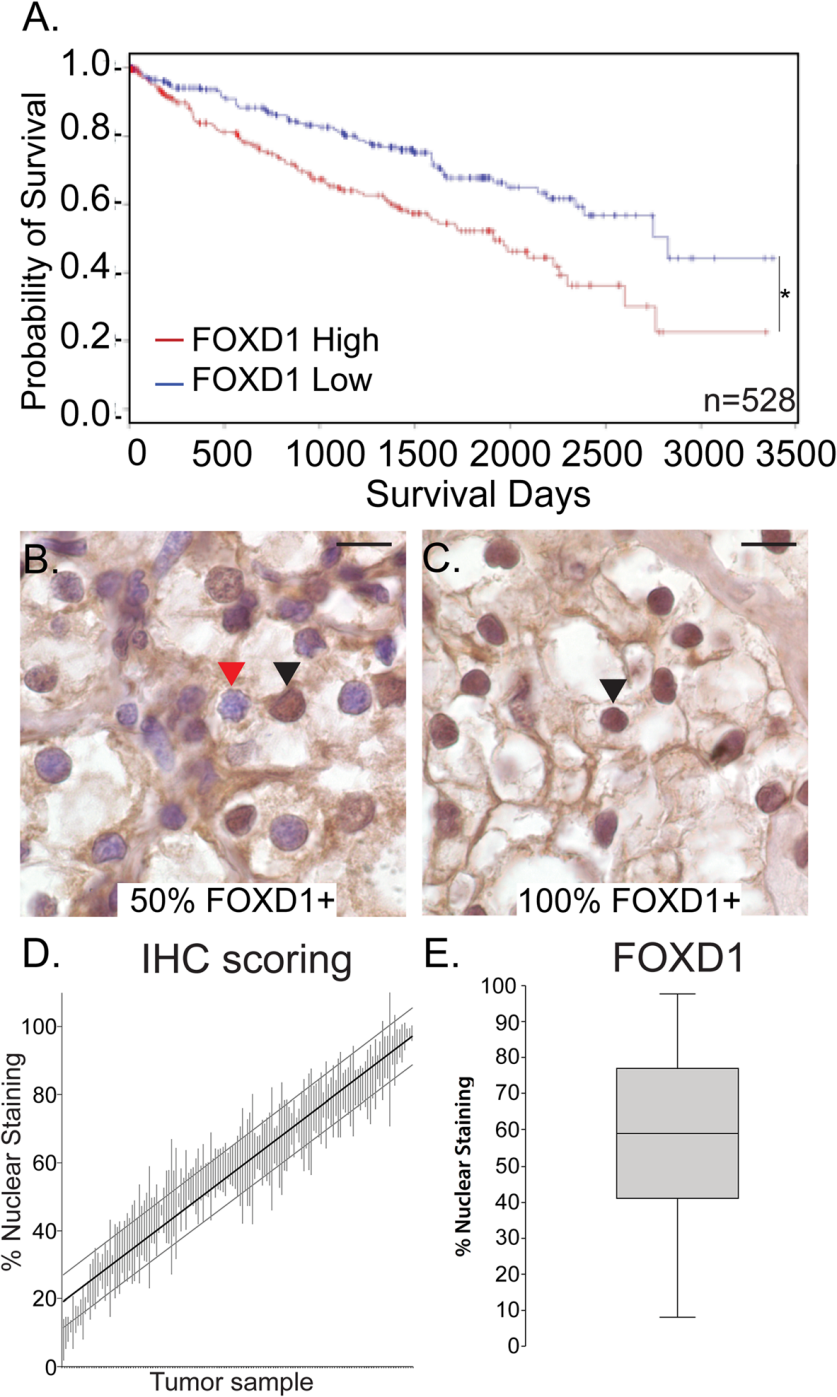
FOXD1 expression in ccRCC. **a** Kaplan-Meier survival analysis for ccRCC patients with high versus low tumor expression of *FOXD1* based on transcriptome data from The Cancer Genome Atlas (TCGA). High and low expression were plotted relative to median expression of FOXD1. *p = 6.85E-05. **b**-**e** FOXD1 expression in ccRCC tumors. Representative examples of tumors with mosaic nuclear staining **b** and ubiquitous nuclear staining **c**. Black arrows indicate positive and red arrow indicates negative nuclei. **d** Scoring of percentage nuclear staining in 142 resected ccRCC tumors. For each tumor, five high power fields were scored for percentage of positive nuclei. **e** The median percentage of FOXD1-positive nuclei in tumors is 58.15% (95% CI 50.6 to 65.7)

To understand if there could be a role for *FOXD1* in ccRCC, we correlated tumor expression with patient outcomes using data from The Cancer Genome Atlas (TCGA). Five hundred and twenty-eight ccRCC cases were grouped into high or low *FOXD1* expression relative to the median. Patients with *FOXD1*-low tumors showed significantly higher probability of survival at all time-points analyzed (Fig. 1a), supporting a role for tumor expression of *FOXD1* in disease progression. Comparison of FOXD1 expression in tumors of different grades showed higher *FOXD1* levels in higher grades (G1 vs. G3 *p* = 1.2 × 10^− 2^; G1 vs. G4 *p* = 5.0 × 10^− 4^; G2 vs. G3 *p* = 3.8 × 10^− 2^; G2 vs. G4 *p* = 1.4 × 10^− 3^) (Fig. S1A). Comparison of FOXD1 expression at different tumor stages revealed a significant difference only between stage 1 and stage 4 (S1 vs. S4 *p* = 1.9 × 10^− 2^) (Fig. S1B). We further investigated if increased *FOXD1* expression could have poor outcomes related to any particular stage and found that high *FOXD1* expression significantly correlated with worse outcomes for patients at stages 2 and 3 (stage 2 *p* = 4.5 × 10^− 2^; stage 3 *p* = 3.3 × 10^− 3^) (Fig. S1C-F).

To define the expression pattern of the FOXD1 transcription factor in ccRCC, we immunostained 142 patient tumors with a histopathological diagnosis of ccRCC (Table S1). Considering the high degree of relatedness between forkhead proteins, cross-reactivity of antisera presents a problem. To identify a FOXD1-specific antibody for these studies, we used *Foxd1* null mice to screen for specificity. *Foxd1* is strongly and specifically expressed in cortical interstitial cells of the developing kidney [4]. Comparison of immunohistochemical staining using several commercial FOXD1 antibodies revealed that the LS Bio FOXD1 antibody (LS-B6453) showed specificity for nuclear FOXD1 in cortical interstitial cells of the developing kidney and did not stain *Foxd1* null tissue (Fig. S2A,B). In immunohistochemistry, diffuse staining is seen in epithelial tubules of adult human kidney tissue (Fig. S2C), and immunohistochemical analysis of *Foxd1* null mouse tissue reveals a similar pattern (Fig. S2D), indicating that this is antibody trapping. For this reason, we limited our analysis to nuclear staining. Nuclear FOXD1 was observed in all tumors (Fig. 1b ,c), with extensive inter-tumor variability in proportion of positive nuclei (Fig. 1d). Taking into account the variability of scores between and within samples, we generated a 95% confidence interval for proportion of FOXD1-positive nuclei in tumors as 58.15 +/− 7.55% (Fig. 1e). We note a potential bias in that a fraction of patients (8/142) received neoadjuvant treatment prior to sample collection. As expected, approximately 50% of Stage IV tumors were from treated patients, and any effect of treatment on FOXD1 expression would primarily be seen in this group. However, the proportion of tumors from treated patients overall is approximately 5% and would only constitute a minor effect on the aggregated data if FOXD1 expression is affected by neoadjuvant treatment.

### Effect of *FOXD1* knockout on expression of known transcriptional targets

**Fig. 2:**
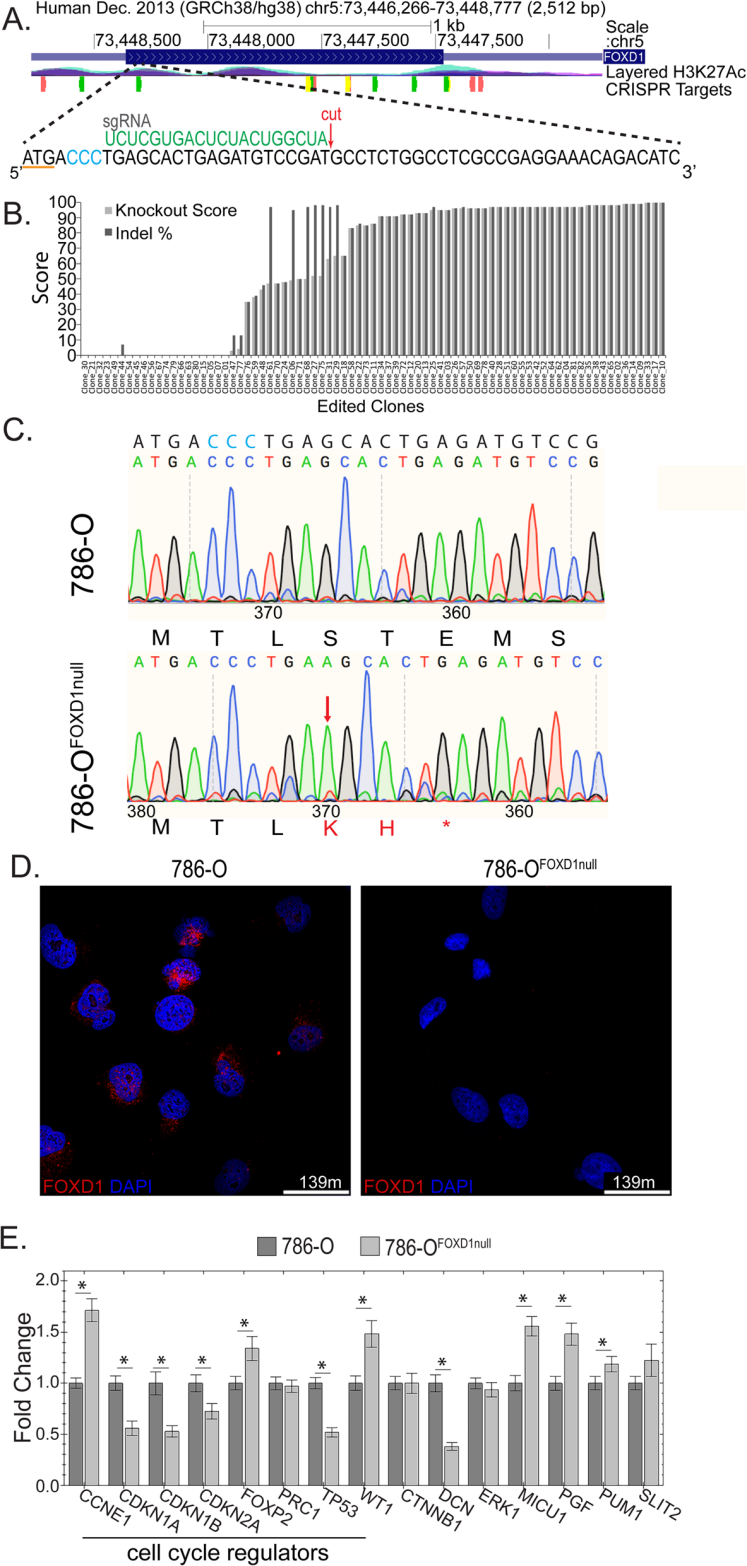
*FOXD1* knockout affects expression of cell cycle regulators and alters cellular energetics. **a** CRISPR/Cas9 knockout targeting strategy for *FOXD1*. The *FOXD1* locus on chromosome 5 shows the single *FOXD1* exon with the CDS in dark blue and UTRs in light blue. The placement of the guide RNA (green) relative to the ATG (underlined) is shown below. **b** Knockout prediction score and percentage of alleles with insertions or deletions (indels) for 82 clones isolated from the CRISPR-edited pool. Chromosome 5 is diploid in 786-O and clones with 50% indel frequency are predicted to be heterozygous, while those with 100% indel frequency are predicted to be homozygous. **c** Sequencing trace of 786-O versus 786-O^FOXD1null^. Red arrow indicates nucleotide insertion. Bottom: Predicted protein sequence showing insertion event resulting in modified amino acid sequence (red text) and premature stop (*). **d** FOXD1 (red) immunofluorescence staining in 786-O versus 786-O^FOXD1null^, counterstained with DAPI (blue). **e** Expression of predicted FOXD1 target genes in edited versus parent cells. *p < 0.05

To understand the function of FOXD1, we generated a loss of function renal cell carcinoma cell line using CRISPR/Cas9. The 786-O cell line, which has a mutation in the *VHL* gene, is diploid for chromosome 5 on which *FOXD1* is located [12] and is therefore a good candidate cell line in which to generate a loss of function. A guide sequence was identified that would cleave the gene 4 codons downstream of the ATG and enable selection of edited clones with insertions or deletions causing truncating mutations (Fig. 2a). Inference of CRISPR Edits (ICE), a regression algorithm for predicting editing outcomes from Sanger sequencing data, was used to predict knockout of *FOXD1* in clones of edited cells (Fig. 2b). Clone 2, with a single thymine insertion at the same position in both copies of *FOXD1*, was predicted to encode a protein that is truncated 4 amino acids downstream of the methionine (Fig. 2c). Sequencing of the top potential off-target genes revealed no unwanted modifications (Table S2). Immunostaining showed an absence of FOXD1 in the nucleus and cell body of Clone 2 (Fig. 2d). We conclude that Clone 2 carries a null mutation in *FOXD1* originating from a homozygous single nucleotide insertion, and we refer to it hereafter as 786-O^FOXD1null^.

To understand transcriptional effects of inactivating *FOXD1*, we compiled a list of candidate targets from previous reports [9, 13–15]. To ensure accurate measurement of expression by RT-QPCR, we selected reference genes with stable expression between 786-O and 786-O^FOXD1null^. Commonly used reference genes such as *GAPDH*, *HPRT*, *TUBB*, and *TBP* showed very poor stability between these cell lines (Fig. S3A). We selected *RPLPPO*, *B2M*, and *HBB* (Fig. S2B-D), and used the geometric mean of these three assays as the reference value. Target genes associated with the cell cycle were misregulated, with the exception of PRC1, suggesting a cell cycle perturbation (Fig. 2e). *DCN*, *PGF*, *PUM1*, and *MICU1* were also misregulated in 786-O^FOXD1null^.

### FOXD1 influences cellular energetics, but does not affect overall ATP production

**Fig. 3:**
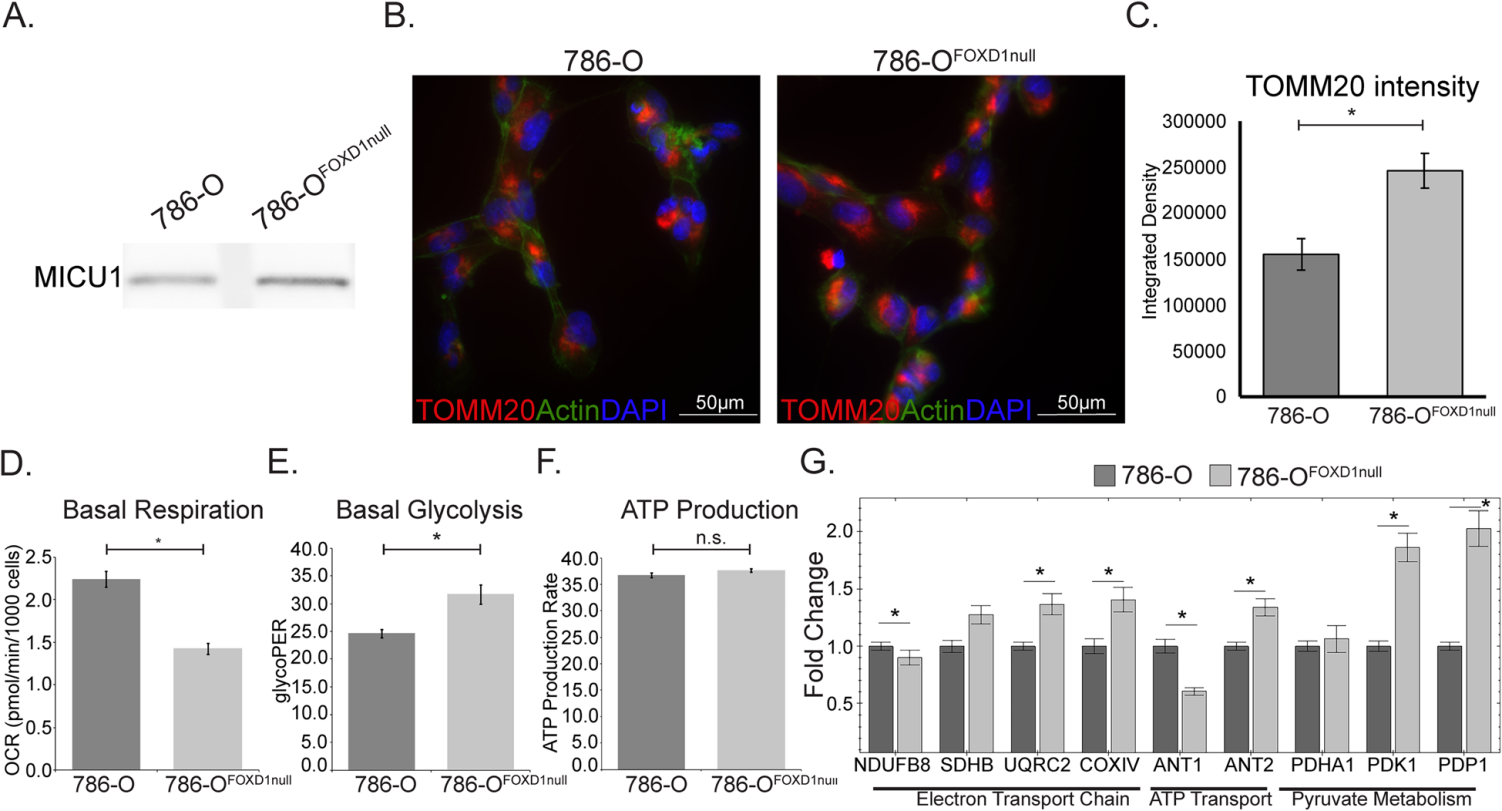
FOXD1 influences mitochondria levels, but does not promote glycolysis. **a** Western Blot analysis of MICU1 in 786-O versus 786-O^FOXD1null^. Loading was normalized by total protein in each lane and the full length blot is available in Supplemental Fig. S7. **b** Immunofluorescence analysis of mitochondria marker TOMM20 (red). Actin filaments (green) used to outline cell body and DAPI (blue) used to outline nucleus (*N* = 2 biological replicates). **c** Quantification of TOMM20 fluorescence intensity between 786-O and 786-O^FOXD1null^ using integrated density. * *p* < 0.05. **d** Basal mitochondrial respiration measured by Seahorse XF mitochondrial stress test (3 biological replicates). **e** Representative basal glycolytic rate comparison via Seahorse XF glycolytic rate assay (2 biological replicates). Y axis values are in pmol/min/1000cells.  **f** Representative total ATP production rate comparison via Seahorse XF real-time ATP rate assay (2 biological replicates). Y axis values are in pmol/min/1000cells. For all Seahorse measurements, each biological replicate was measured in 5 technical replicates. *p < 0.05 **g** Expression of genes involved in mitochondrial respiration in edited versus parent cells. *p < 0.05

Considering that ccRCC is a disease of cellular metabolism, misregulation of *MICU1* is particularly interesting because it affects calcium signaling between cytoplasm and mitochondria [14]. In agreement with previous studies, our data shows that FOXD1 represses *MICU1* transcription [14]. Immunoblot revealed a significant increase in MICU1 protein in the 786-O^FOXD1null^ compared to the parent 786-O (Fig. 3a). To understand if this reflects an increased abundance of mitochondria in the 786-O^FOXD1null^, we immunostained both lines for the mitochondrial marker TOMM20, and found increased signal intensity in 786-O^FOXD1null^, suggesting increased mitochondrial abundance (Fig. 3b, c). To test if this altered utilization of oxidative metabolism, we compared 786-O versus 786-O^FOXD1null^ using Seahorse XF. Surprisingly, we found a significant reduction in basal mitochondrial respiration in 786-O^FOXD1null^ (Fig. 3d), and an increase in basal glycolysis (Fig. 3e). However, total ATP production between the lines was equivalent (Fig. 3f), indicating that energy derived from increased glycolysis compensates for the decrease in mitochondrial respiration. Thus, despite elevated MICU1 expression and evidence of increased mitochondrial abundance, mitochondrial respiration is depressed in the 786-O^FOXD1null^. The 786-O cell line has a *VHL* inactivating mutation and is constitutively hypoxic with elevated baseline expression of HIF2. Persistent expression of HIF transcription factors represses respiratory chain subunits [16], and we therefore analyzed expression of genes in the mitochondrial respiration pathway (Fig. 3g). Modest changes were found for electron transport chain components, with *UQRC2* and *COXIV* showing elevated expression in 786-O^FOXD1null^. The ATP transporters *ANT1* and *ANT2* both showed significant changes, but in opposite directions making the net effect unclear. Expression of genes encoding pyruvate dehydrogenase kinase 1 (*PDK1*) and pyruvate dehydrogenase phosphatase catalytic subunit 1 (*PDP1*) was significantly elevated. PDK1 and PDP1 act in opposition on the enzyme pyruvate dehydrogenase, which is essential for energy production from the TCA cycle. Their simultaneous upregulation in the 786-O^FOXD1null^ suggests that elevated PDK1 may inhibit the TCA cycle, reducing mitochondrial respiration [17], with concomitant upregulation of the PDP1 phosphatase as a feedback response. This would be predicted to result in aerobic glycolysis being favored over mitochondrial metabolism in the 786-O^FOXD1null^ cell. In summary, although mitochondria may be more abundant in 786-O^FOXD1null^, our data supports reduced mitochondrial energy production, and our gene expression analysis suggests inhibition of the TCA cycle.

### FOXD1 is essential for tumor growth in vivo

**Fig. 4:**
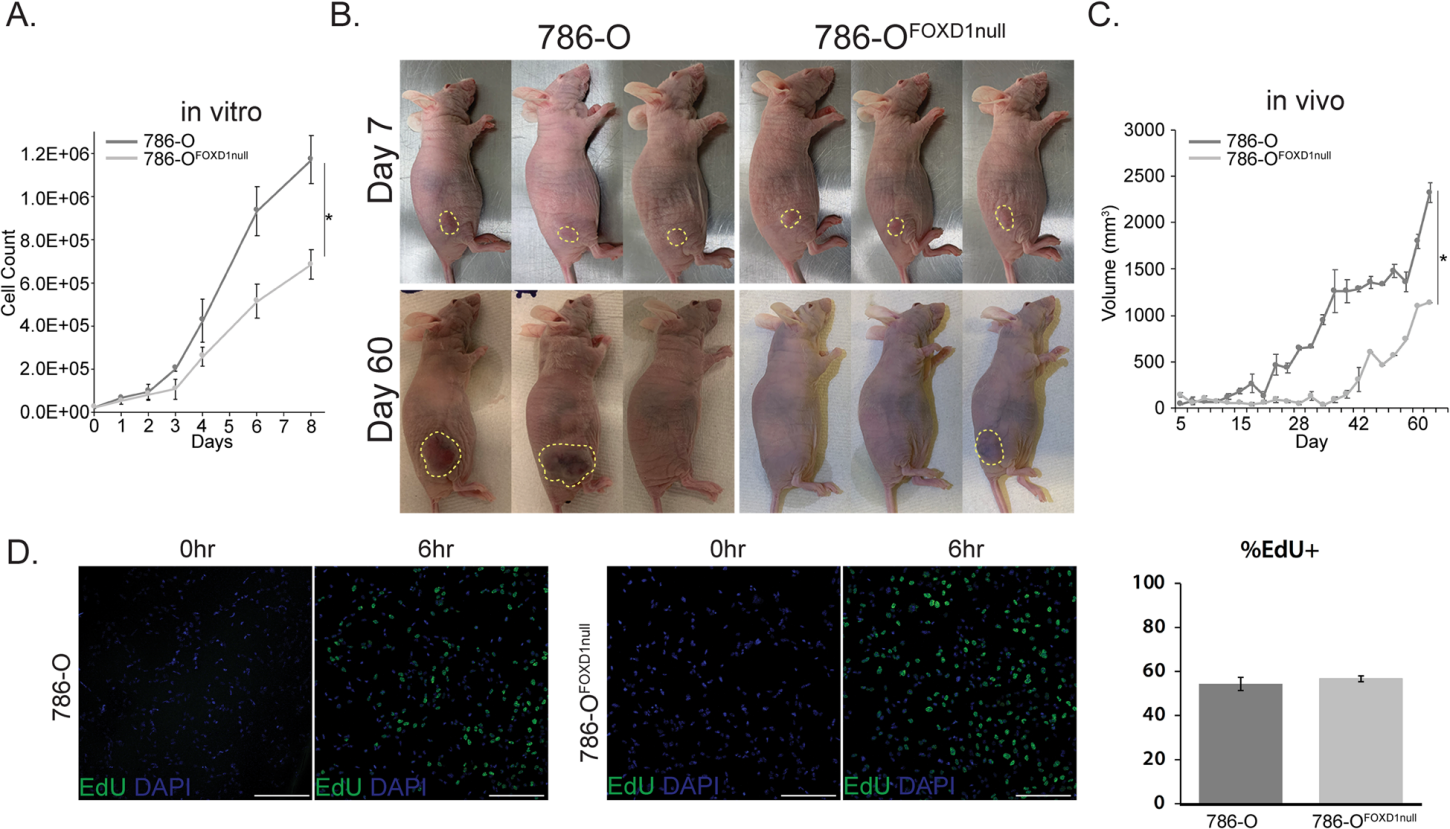
Loss of FOXD1 reduces tumor growth in vitro and in vivo. **a** In vitro growth curves for 786-O and 786-O^FOXD1null^. * *p* < 0.05 **b** Xenografts of 786-O versus 786-O^FOXD1null^ cells in nude-beige mice. Dotted yellow lines outline tumors on days 7 and 60 after injection. **c** Xenograft growth rate analysis. * *p* < 0.05 **d** 786-O and 786-O^FOXD1null^ cells pulsed with EdU for 2 h and chased for 4 h. The percentage of all cells,stained with DAPI (blue) positive for EdU (green) is shown in the bar chart (*N* = 3)

Although total ATP production is unaffected by inactivation of *FOXD1*, proliferation is attenuated in 786-O^FOXD1null^ (Fig. 4a), suggesting that effects on the cell cycle (Fig. 2e) may be primary rather than a consequence of energy deficit. To understand if the effect of FOXD1 on cellular proliferation is reflected in a tumor model, we xenografted cohorts of 6 nude mice with either 786-O or 786-O^FOXD1null^ (Fig. 4b) as previously described [18, 19]. After tumor establishment, 70% of 786-O^FOXD1null^ tumors regressed, compared to only 30% for 786-O (Fig. S4A,B). 786-O^FOXD1null^ tumors that were established showed significantly reduced growth rate compared to 786-O (Fig. 4c). FOXD1 promotes expression of CDKN1A/p21 [20], and our expression analysis of 786-O^FOXD1null^ showed reduced *CDKN1A/p21* expression (Fig. 2e). CDKN1A/p21 delays the G1 to S transition, and reducing its expression is predicted to accelerate cycling time, which is contradictory to our observations in vitro and in vivo. To investigate if *FOXD1* inactivation influences the G1-S transition, we performed an EdU pulse-chase experiment to analyze the percentage of cells able to enter S phase. A 2 h EdU pulse followed by a 4 h chase did not show any difference in S phase entry between 786-O and 786-O^FOXD1null^ (Fig. 4d), and it is unlikely that the reduced proliferation in *FOXD1* null cells is due to delayed G1-S transition.

### Loss of FOXD1 causes cell cycle delay at G2/M and inability to phosphorylate histone H3

**Fig. 5:**
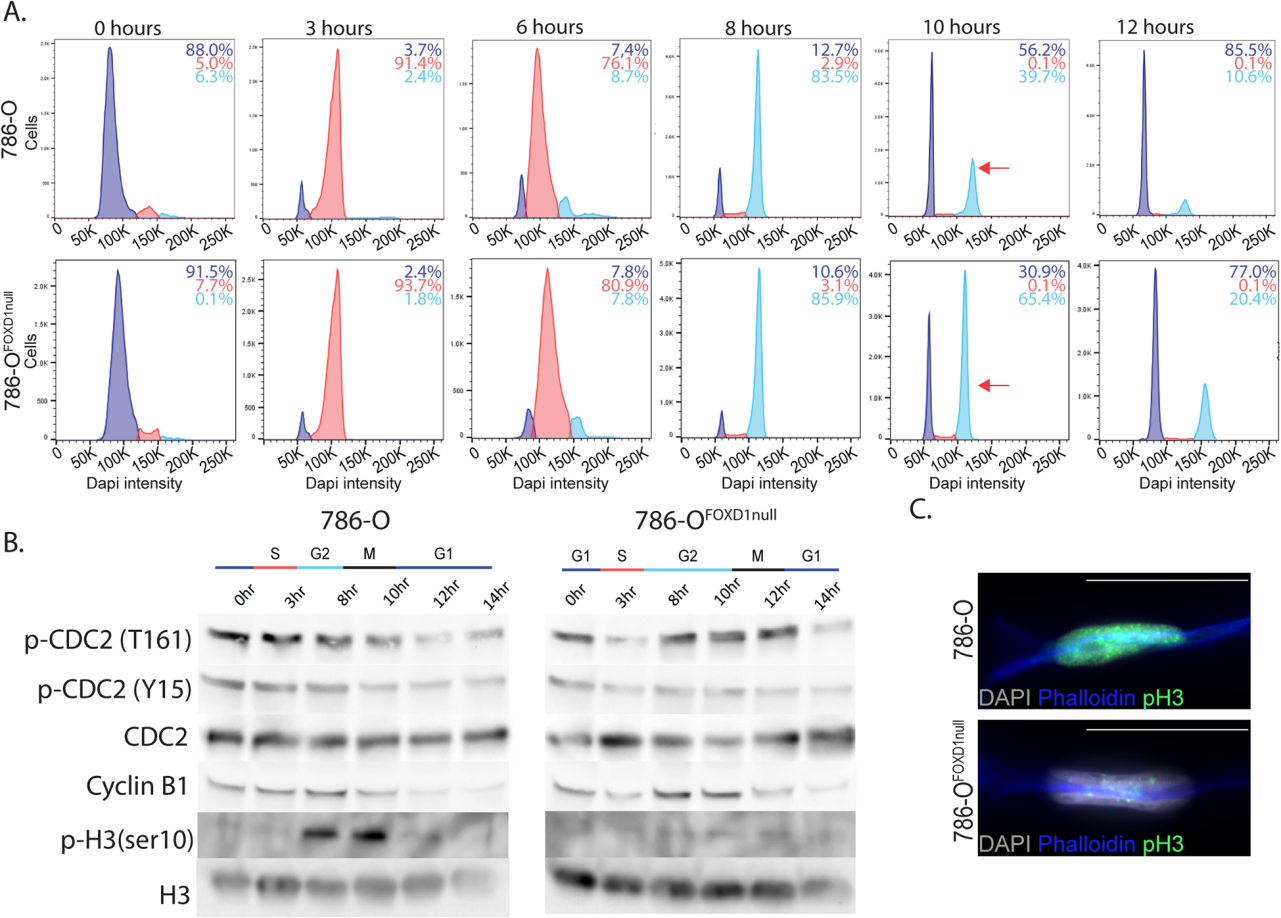
G2 progression and histone H3 phosphorylation at ser10 are impaired in FOXD1 null cells. **a** Cell cycle analysis of synchronized cells at intervals after release from double thymidine block. Red arrows indicate a significant difference (*p *< 0.05) in phase distribution between lines after 10 h post-block (*N* = 2). **b** Western blot analysis of proteins necessary for progression through G2/M at intervals after release from synchronized block, comparing 786-O and 786-O^FOXD1null^. Predicted cell cycle phases at each time point are graphically represented above the blots. Full-length blots are presented in Supplementary Figs. S5, S6, and S7. **c** Representative pH3 (green) immunocytochemistry of 786-O parent and 786-O^FOXD1null^ of cells in metaphase, counterstained with phalloidin (blue) and DAPI (gray)

To determine if the reduced proliferation caused by loss of *FOXD1* may be due to stalling at the G2/M checkpoint, we synchronized cells with a double thymidine block and timed phases of the cell cycle by analyzing DAPI incorporation (Fig. 5a). Corroborating the EdU analysis, both lines proceed at the same rate from G1 to S phase and by 8 h both have entered G2/M. While 786-O cells have largely transitioned out of M by 10 h, the transition of 786-O^FOXD1null^ out of M is delayed by 2–4 h. G2/M delay provides an explanation for the reduced growth of 786-O^FOXD1null^ in tumor xenografts and in vitro.

To further understand when cells pass the G2/M checkpoint, we investigated a panel of markers selected as follows. CDC2 and CyclinB1: The activated CDC2-CyclinB1 complex is necessary for progression through the G2/M checkpoint into mitosis [21]. CyclinB1 steadily rises through the early stages of the cell cycle until peaking at late G2, after which its level rapidly declines. CDC2, when phosphorylated on T161, activates the CDC2-CyclinB1 complex and initiates progression through the G2/M checkpoint. However, phosphorylation at Y15 inactivates the complex and prevents progression through the checkpoint. In order for cells to proceed into mitosis, CDC2 would need phosphorylation at T161 and dephosphorylation at Y15. In 786-O cells, phosphorylation of CDC2 occurs at both T161 and Y15 until 10 h, at which time phosphorylation of Y15 significantly decreases while T161 levels are still elevated. By 12 h, phosphorylation at T161 significantly drops, indicating completion of mitosis. Additionally, CyclinB1 levels steadily increase until 8 h before rapidly dropping at 10 h. This supports our previous prediction that cells enter G2/M by 8 h post-synchronization, and complete mitosis by 12 h post-synchronization. In comparison, the 786-O^FOXD1null^ cells maintain elevated p-CDC2(T161) levels through the same time course, only reducing levels at 14 h. p-CDC2(Y15) levels in parallel showed constitutively high levels until 12 h post-synchronization. CyclinB1 peaks at 8 h, like the parental line, but this level is maintained until 12 h post-synchronization. Serine 10 phosphorylation of histone H3 at promoter regions of select genes such as *FOS* and *JUN* [22] regulates abundance of the AP1 transcription factor that is required for expression of mitotic regulators including Aurora kinase B [23]. Because of the abundance of this phosphorylation, it is commonly used as a marker of mitosis. We thus chose to look additionally at pH 3 and found that 786-O^FOXD1null^ showed significantly reduced levels of pH3 compared to 786-O at the G2/M-G1 interface (Fig. 5b, Fig. S5). Despite abundant expression of histone H3 in 786-O^FOXD1null^, phosphorylation is very modest compared with 786-O.

Cells were immunostained to understand if the phosphorylation of H3 is reduced in all cells, or if there are fewer cells with H3 phosphorylation in 786-O^FOXD1null^ compared with 786-O. All nuclei in 786-O^FOXD1null^ showed depressed phosphorylation of H3. Phosphorylation of H3 peaks at metaphase, a stage that is defined histologically by colocalization of actin fibers with chromosomes. Comparing nuclei of 786-O with 786-O^FOXD1null^ at this stage revealed little pH3 in the 786-O^FOXD1null^ (Fig. 5c, Table S4). These findings support a requirement for FOXD1 in pan-chromosome phosphorylation of H3 serine 10. Overall, loss of FOXD1 results in prolonged time spent at the G2/M checkpoint and an inability to phosphorylate histone H3.

### FOXD1 expression is necessary for genomic integrity through mitosis

**Fig. 6:**
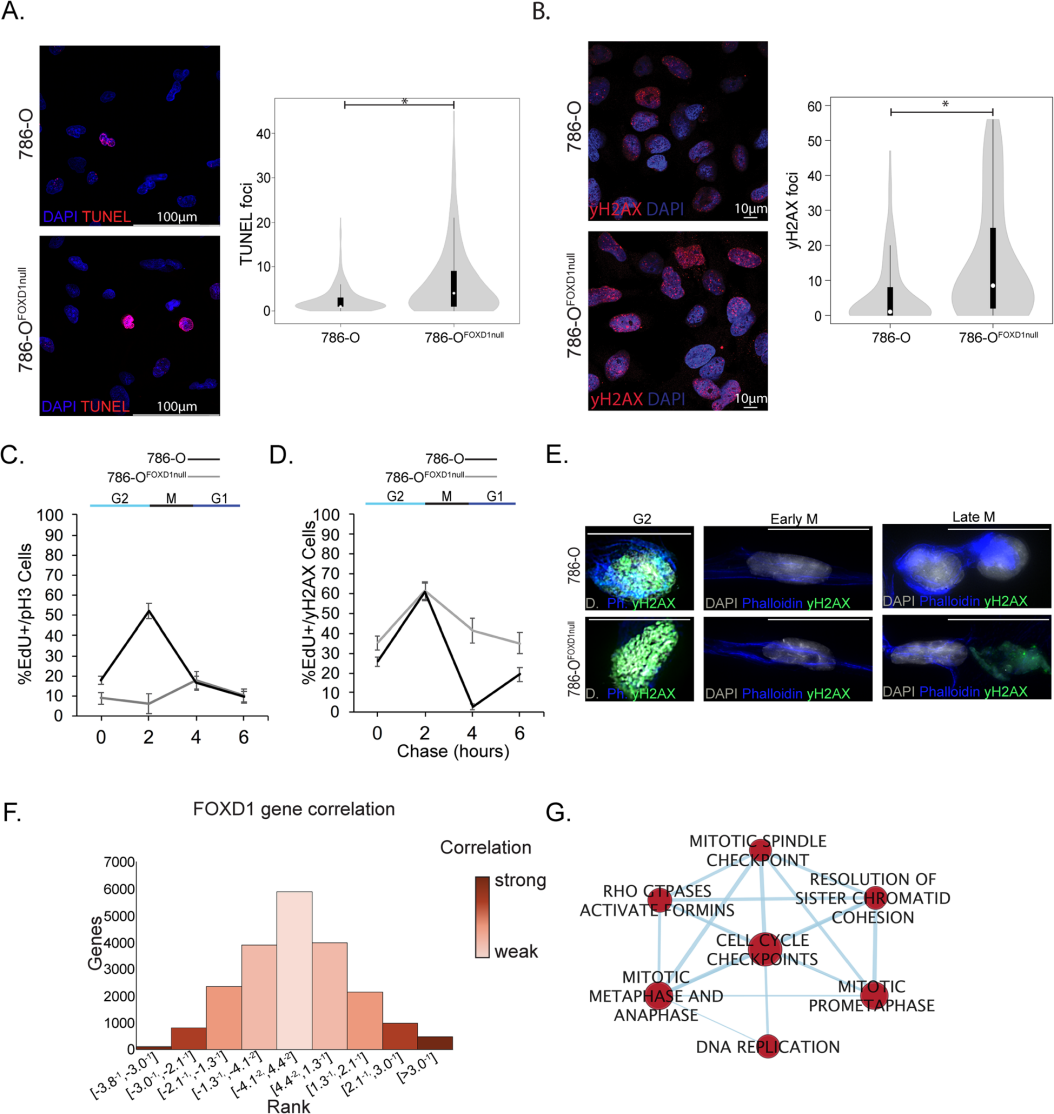
FOXD1 is necessary for maintaining DNA integrity following mitosis. **a** Representative TUNEL (red) staining of 786-O versus 786-O^FOXD1null^, counterstained with DAPI (blue) . Nuclear TUNEL foci were counted and compared using Student's t-test (*N* = 3). *p < 0.05 **b** Representative γH2AX (red) immunocytochemistry of 786-O versus 786-O^FOXD1null^ cells, counterstained with DAPI (blue). Nuclear γH2AX foci were counted and compared using Student’s t-test (*N* = 3). *p < 0.05 **c** EdU pulse-chase cell cycle progression analysis. Cells in G2 were labeled with EdU following 8 h incubation with EdU. Labeled cells were analyzed for pH3 expression every 3 h post-labeling using immunocytochemistry. One hundred EdU+ cells were analyzed (*N* = 3). **d** Analysis of EdU labeled cells in G2 for γH2AX expression every 2 h post- labeling by immunocytochemistry. One hundred EdU+ cells were analyzed (*N* = 3). **e** Immunocytochemistry analysis of γH2AX (green) at different phases of the cell cycle, costained with DAPI (gray) and phalloidin (blue). Early M represented by cells in metaphase and late M representing cells undergoing cytokinesis and entering post-mitosis G1. **f** Correlation of mean FOXD1 expression against all genes found in the KIRC TCGA database. Ranked values scored using the Pearson correlation coefficient. **g** Gene set enrichment analysis of ranked FOXD1-correlated genes shows enrichment of REACTOME pathways related to mitotic entry and progression

A second essential function of histone H3 phosphorylation is the condensation of chromosomes, which depends on serine 10 phosphorylation of H3 throughout each chromosome [24]. Improper chromosome condensation promotes DNA damage [25], and we therefore compared strand breaks in 786-O with 786-O^FOXD1null^ by staining for TUNEL and found that 786-O^FOXD1null^ cells show a significantly increased numbers of TUNEL-positive foci per nucleus than 786-O (Fig. 6a). Each TUNEL puncta represents DNA strand breakage, and the analysis suggests that only approximately 50% of 786-O^FOXD1null^ cells have intact genomes compared with 80% of 786-O. In response to DNA damage, cells typically phosphorylate H2AX (γH2AX) at locations of double strand breaks to indicate regions needing DNA repair. We further investigated if γH2AX levels were also elevated in 786-O^FOXD1null^ cells, and found a similar pattern to TUNEL, although the proportions of nuclei staining with γH2AX was greater, suggesting that this assay is more sensitive (Fig. 6b). DNA damage is normally repaired during G2/M and cells that have undergone mitosis should display strongly reduced γH2AX staining. To understand if DNA damage is repaired during G2, we performed an EdU pulse-chase experiment. Although synchronizing cells as shown in Fig. 5a would facilitate this experiment, the synchronization procedure introduces DNA damage, and would confound the analysis. Based on an S phase length of approximately 6 h (Fig. 5a), we performed an 8 h pulse to label cells entering G2 (Fig. S6). Expression of pH3 in 786-O showed a peak at 2 h following EdU wash-out and defined a subpopulation that had synchronously transitioned to mitosis (Fig. 6c). By 4 h after wash-out, the number of pH3+ cells had returned to baseline, indicating exit of this subpopulation from mitosis. Immunostaining for γH2AX in this subpopulation revealed that staining peaked concomitantly with pH3 and declined to 0% 4 h after wash-out (Fig. 6d), showing a complete reset of the chromosomal markers for DNA damage and suggesting comprehensive DNA damage repair in 786-O. As anticipated, 786-O^FOXD1null^ did not show a pH3 peak (Fig. 6c), but γH2AX immunostaining of the pH3+ subpopulation did show a peak at 2 h after wash-out (Fig. 6d). However, γH2AX declined only modestly following the peak, showing that chromosomal damage markers were not reset, and suggesting remaining DNA damage following mitosis in 786-O^FOXD1null^. These findings were replicated with 3 different EdU-pulse labeling time periods (Fig. S6). To determine if DNA damage was reduced in all 786-O^FOXD1null^ cells, or if the 786-O culture may be a mosaic of cells in which DNA damage is repaired and cells in which it is not, we compared nuclei of 786-O and 786-O^FOXD1null^ cells immunostained with γH2AX, phalloidin, and DAPI to define mitotic cells with colocalized chromatin and actin (Fig. 6e). Interestingly, while γH2AX was not detected in cells going through anaphase in either 786-O or 786-O^FOXD1null^, we found a significant number of cells with γH2AX in at least one daughter cell during cytokinesis, indicating that the sustained DNA damage found in 786-O^FOXD1null^ is likely due to strand breaks that arise during mitosis (Table S4).

Taken together, our data shows that *FOXD1* is required for 786-O tumor cells to undergo mitosis with DNA damage repair of both daughter cells. To understand if this mechanism could underlie the reduced patient survival seen in ccRCC cases with high *FOXD1* tumor expression (Fig. 1a), we performed a correlation analysis between *FOXD1* expression in ccRCC tumors and biological pathways. First, *FOXD1* expression was correlated with expression of all other genes in ccRCC transcriptomes from the TCGA database (Fig. 6f). Strongly correlated genes were then analyzed using Gene Set Enrichment Analysis to identify biological pathways (Fig. 6g). *FOXD1* expression was associated with cell cycle checkpoint pathways and pathways controlling chromosomal architecture in mitosis, suggesting that FOXD1 may indeed participate in these processes in patient tumors. The top common pathway components include members of the nuclear pore complex (*SEC13, NUP37)*, centromere subunits (CENPM, CENPN, CENPQ), and enzymes (PPP2R5A, PPP2R5B, CDC20, BUB1). Candidate genes within the top scoring pathways include kinases and phosphatases involved in phosphorylation of Histone H3 (Fig. S7).

## Discussion

Our analysis indicates that FOXD1 is required for the appropriate phosphorylation of histone H3, which maintains DNA integrity during mitosis. The capacity for DNA damage repair differs widely between different types of tumors, and ccRCC displays moderate genomic damage [26]. ccRCC is generally resistant to both cis-platinum and radiation-induced DNA damage, suggesting active DNA damage repair in these tumors. Our data suggests that FOXD1 expression is necessary for proper cell division and may serve a protective role during mitosis in tumor cells predisposed to severe genetic damage.

Based on previous work demonstrating a role for FOXD1 in controlling expression of MICU1, which regulates calcium flux in the mitochondrion, we hypothesized that reduced proliferative capacity of *FOXD1* null tumor cells may be due to reduced energy production capacity [14]. Metabolic analyses revealed a bioenergetic shift away from mitochondrial respiration but also demonstrated compensation by increased glycolysis resulting in unaltered ATP production. In light of this finding, reduced energy production does not explain the reduced proliferative capacity. Despite maintained energy production, a shift towards glycolysis may have consequences for basic cell behaviors through the production of a range of metabolites for biosynthetic pathways, and in other tumor cell contexts this is characteristic of increased proliferation [27]. Thus, it is counterintuitive that the bioenergetic shift seen in *FOXD1* null cells would explain reduced proliferation, and the cause is more likely to be a direct effect on the cell cycle.

The loss of histone H3 phosphorylation seen upon *FOXD1* inactivation does not prevent entry into mitosis, and compensatory mechanisms, such as CENPA nucleosome formation, may allow mutant cells to complete the cell cycle [28]. We found centromere subunits associated with this complex were highly correlated with FOXD1 expression, further supporting this possibility (Fig. 6g). However, the CENPA nucleosome complex is known to be unstable in vivo and this compensatory mechanism may be limited to cell culture [29, 30], providing an explanation for the difficulty in generating tumors from 786-O^FOXD1null^ xenografts.

*FOXD1* expression is tightly controlled in the adult, and our analyses of healthy human kidney tissue showed no expression in tubule epithelium, which is the cell population of origin for ccRCC [31]. *Foxd1* was initially characterized as a regulator of kidney development through studies in knockout mice [4], and it is specifically expressed in the progenitor population of the kidney interstitium [32]. Interestingly, recent gene expression analysis has shown a slightly broader expression pattern for *FOXD1* in the human fetal kidney, including a subset of progenitor cells of the tubule epithelium [33]. Thus, the expression of FOXD1 in dedifferentiated tubule epithelial cells in ccRCC may represent partial regression to a developmental progenitor for the tubule epithelium. The nephron progenitor cell population of the developing kidney is highly proliferative, and studies in mouse suggest that it relies heavily on glycolysis, similarly to the ccRCC cell [34]. *FOXD1* is located on chromosome 5 approximately 60 megabases from *VHL*, which is the most common primary mutation in ccRCC. Thus, an alternate possibility is that *FOXD1* expression becomes deregulated by the genetic hit at the *VHL* locus that initiates transformation, rather than being activated as a component of a dedifferentiation program. 

The finding that FOXD1 expression is mosaic in the majority of tumors indicates that it may be activated or inactivated in subpopulations of tumor cells. Given its role in promoting cell cycle progression and DNA damage repair, *FOXD1* would be expected to confer a growth advantage, and for this reason it seems most likely that it is activated in subsets of cells as the tumor ages. Mechanisms governing cellular localization of FOXD1 are not known, and an alternate explanation for the mosaicism that we see in patient tumors may be that nuclear FOXD1 accumulation is dynamic and regulated by environmental factors that differ in distinct regions of tumors. Structure-function studies of the FOXD1 protein and a better understanding of tumor architecture will be required to evaluate these possibilities.

This study uncovers a novel and important role for FOXD1 in controlling division and DNA repair of ccRCC tumor cells. Further studies aimed at defining how the activity of FOXD1 is regulated will determine if this is a tractable therapeutic pathway.

## Conclusions

This work supports a role for FOXD1 as a potent driver of tumor growth in ccRCC. *FOXD1* expression inversely correlated with patient outcome and was also shown to be grade and stage dependent. Inactivation of *FOXD1* significantly decreased the expression of components of mitochondrial metabolism in ccRCC cells and promoted glycolysis, but did not influence overall energy production. The cell cycle was altered by loss of *FOXD1*, with a delay in progression through the G2/M checkpoint, which associated with increased DNA damage. Phosphorylation of histone H3 was lost in *FOXD1* null cells, providing an explanation for the increase in mitotic defects. Based on our investigation, we propose that FOXD1 is required for histone H3 phosphorylation in rapidly cycling ccRCC cells, ensuring that they can proceed through the cell cycle without catastrophic DNA damage. Transcriptomic evidence from human ccRCC tumors correlating *FOXD1* expression with expression of kinases and phosphatases that modulate histone H3 phosphorylation supports this interpretation. Understanding how FOXD1 controls mechanisms governing histone H3 phosphorylation in ccRCC has potential therapeutic applications that will be explored in future work.

## Supplementary Information

**Additional file 1 Table S1:** Summary of patient clinical data used for FOXD1 immunohistochemistry analysis. **Table S2:** Primers used for FOXD1 genotyping and off-target analysis. * Blue indicates primers used for sequencing mismatch region. ** Red bases indicate mismatches from FOXD1. **Table S3**: Primers for reference genes, FOXD1 targets, and mitochondrial metabolism components. **Table S4**: Nuclear morphology analysis of 786-O and 786-O^FOXD1null^. *Immunocytochemistry analysis of cells in different phases of the cell cycle after low density plating, based on nuclear and actin morphologies and localization. Percentage values are based on analysis of 100 cells per group across several fields. **Defects panel indicates collection of possible mitotic defects including lack of chromosome condensation (prophase), loss of spindle polarity (metaphase/anaphase), incomplete sister chromosome separation (cytokinesis), and death of daughter cell (post-mitosis G1). **Figure S1**: FOXD1 grade and stage analyses. (A) *FOXD1* expression level comparisons at different ccRCC tumor grades based on transcriptome data from The Cancer Genome Atlas (B) *FOXD1* expression level comparisons at different ccRCC tumor stages based on transcriptome data from The Cancer Genome Atlas. (C-D) Kaplan-Meier survival analyses for ccRCC patients with high versus low tumor expression of *FOXD1*, analyzed based of tumor stage. *p < 0.05. **Figure S2**: FOXD1 antibody validation. (A-B) TSA amplification staining for FOXD1 (green) on E12.5 mouse kidneys on normal and FOXD1-null backgrounds. Nuclei counterstained with DAPI (blue), (C-D) Immunohistochemistry staining of adult human kidney tissue (C) and FOXD1-null E12.5 mouse kidney (D). **Figure S3**: FOXD1 qPCR reference gene selection. (A) Stability scores generated by comparing candidate reference gene assays (Table S3) on equivalent mRNA quantities of 786-O versus 786-O^FOXD1null^ analyzed using the BioRad Reference Gene Selector Tool. Colors of bars denote genes that are over (green) or under the acceptable stability threshold between samples. (B-D) Standard curves for reference genes selected for this study. **Figure S4**: FOXD1-null cells difficulty in tumor establishment. (A) Xenograft of 786-O and 786-O^FOXD1null^ into flanks of 6-week old NCG mice. Red dotted line outlines tumors. Resected tumors from shown mice are shown in right-hand corner of image (B) Xenograft growth rate analysis. **Figure S5**: Histone H3 phosphorylation analysis. (A-B) Full membranes used for Western Blot analysis, with proteins visualized using the Bio-Rad Stain-Free gel system. Protein loading was quantified in each lane for normalization. (C-F) Full protein blots for histone H3 and phosphorylated histone H3 (Ser10) of synchronized 786-O and 786-O^FOXD1null^ cells at designated time points after release from thymidine block. Blue boxes indicate areas used for quantification. (G) Densitometry analysis (Ser10), normalized to total protein in well. **Figure S6**: CDC2 Phosphorylation analysis. Full membranes and quantification used for western blot analysis of CDC2 (A-B) and phosphorylated forms at T161 (C-D) and Y15 (E-F). All protein levels were normalized to total protein loaded. **Figure S7**: CyclinB1 and MICU1 protein analysis. Full membranes and quantification used for western blot analysis of CyclinB1 (A-B) and MICU1 (C-D). All protein levels were normalized to total protein loaded. **Figure S8**: EdU pulse-chase analysis. (A) Labeling schematic of pulse-chase experiment. Cells in S phase incorporate EdU and are labeled (yellow circles). After 8 h EdU treatment, cells in G2 are labeled with EdU. After 2 h (2 h chase), labeled cells in G2 progress into mitosis (M). After an additional 2 h (4 h chase), labeled cells in M divide and enter G1. (B) Representative images showing staining of pH 3 and γH2AX at 2 h intervals following EdU pulse of both 786-O and 786-O^FOXD1null^. (C) EdU pulse-chase cell cycle progression analysis. Cells in G2 were labeled with EdU following 6 h or 10 h incubation with EdU. Labeled cells were analyzed for pH3 expression every 2 h post-labeling using immunocytochemistry. One hundred EdU+ cells were analyzed (*N* = 3). (D) Analysis of EdU labeled cells, for 6 h or 10 h inncubations, for γH2AX expression every 2 h post- labeling by immunocytochemistry. One hundred EdU+ cells were analyzed (*N* = 3). **Figure S9**: FOXD1 correlated genes involved in cell cycle regulation. Leading edge analysis of FOXD1 correlated genes from TCGA and top scoring candidates. *indicates kinases linked to phosphorylation of histone H3.

## Data Availability

The datasets analyzed during the current study are available in the TCGA repository, https://portal.gdc.cancer.gov/projects/TCGA-KIRC. All data analyzed during this study are included in this published article. All datasets used and analyzed during the current study are available from the corresponding author on reasonable request.

## Abbreviations

TCGA: The Cancer Genome Atlas
ccRCC: Clear cell renal cell carcinoma
ICE: Inference of CRISPR Edits
H3: Histone H3
pH 3: Phosphorylated histone H3
