# Supplementary material for: FOXD1 regulates cell division in clear cell renal cell carcinoma
Source: BMC Cancer. 2021 Mar 24;21:312. doi: 10.1186/s12885-021-07957-8 (PMC7988646; doi:10.1186/s12885-021-07957-8)

| **Sex** | **Male** | **Female** | **All** |
| --- | --- | --- | --- |
| **Count** | 82 (57.7%) | 60 (42.3%) | 142 |
| **Age** | | | |
| Mean | 61.13 | 62.67 | 61.78 |
| Median | 61 | 62 | 62 |
| **Stage** | | | |
| I | 9 | 13 | 22 |
| II | 38 | 25 | 63 |
| III | 19 | 13 | 32 |
| IV | 4 | 5 | 9 |
| **Unknown** | 11 | 4 | 15 |
| **Grade** | | | |
| 1 | 39 | 38 | 77 |
| 2 | 9 | 10 | 19 |
| 3 | 22 | 9 | 31 |
| 4 | 9 | 3 | 12 |
| **Unknown** | 3 | 0 | 3 |
| **Overall Survival** | | | |
| **Mean (S.D.)** | 67.02(±54.37) | 77.12 (±54.35) | 71.30(±53.75) |
| **Median** | 51 | 65 | 58.5 |
| **Recurrence** | 6(8.5%) | 3(5.0%) | 10(7.0%) |

| Gene | Chr. | Ploidy | Cut Site | Sequence | PAM | Forward Primer | Reverse Primer |
| --- | --- | --- | --- | --- | --- | --- | --- |
| FOXD1 | 5 | 2N | 73448353 | ATCGGACATCTCAGTGCTCA | GGG | GAGCTCTGTTCTTAGACTCTCACC | ATAGTGATGAGCGCGATATACGAG * |
| CCR3 | 3 | 4N | 46212264 | ATCTCACATCTCTGTGATCA** | CGG | TGGCCCTGTTTCTGGTTCTCCT | GGTGGAAAGAGAGGCTGGGGAA |
| KCNMB2 | 3 | 4N | 178490962 | ATCAGACATTTCAGTGCTCT | TGG | ACATAGGTGGGTGCTCAACAGA | AGCTGCACTGTGAGCTGTTCTC |
| KIAA0930 | 22 | 3N | 45216858 | CTCGGACAGCTCTGTGCTCA | CGG | ACCTGCCTTAGCGTTCCAAGGT | AGGTTTTCCCCACCCTCAAGCA |
| PRMT3 | 11 | 2N | 20450963 | ATAGTACATCTTATTGCTCA | GGG | ATACCGACGTAGAGTTGCCGGC | GCCACTACTGGTGTCACAAATACGC |
| Chr1 14323846 | 1 | 4N | 114323846 | CCTGGACATCAGTGCTCT | GGG | TGCAGCAGTAAGTGTAAGCCCT | GAGGCTGTGGGTGTTGAGGAGT |
| Chr2 14141430 | 2 | 4N | 10294387 | AACCCACATCTCAGTACTCA | TGG | TGACTGCCTTTGGTGGTCAGCT | AGTGTTCTCCCCACACACACAA |
| Chr10 105657961 | 10 | 3N | 105657961 | ATGGTACTTCTCAGTGCACA | AGG | CCCATTTGCCTGTGTAAAAGTTGGG | AGAGCCTAGCAGACTGTGTGGC |

| **Target** | **Forward Sequence** | **Reverse Sequence** |  |
| --- | --- | --- | --- |
| **Reference Gene Panel** | | | |
| RPLPPO | TGG TCA TCC AGC AGG TGT TCG A | ACA GAC ACT GGC AAC ATT GCG G |  |
| B2M | CCA CTG AAA AAG ATG AGT ATG CCT | CCA ATC CAA ATG CGG CAT CTT CA |  |
| TFRC | ATC GGT TGG TGC CAC TGA ATG G | ACA ACA GTG GGC TGG CAG AAA C |  |
| HBB | CAC CTT TGC CAC ACT GAG TGA G | CCA CTT TCT GAT AGG CAG CCT G |  |
| PUM1 | GCA TTT GGA CAA GGT CTG GCA G | GCT ACA AGT CGA ACA GGA GCT C |  |
| LDHA | GGA TCT CCA ACA TGG CAG CCT T | AGA CGG CTT TCT CCC TCT TGC T |  |
| ACTB | CAC CAT TGG CAA TGA GCG GTT C | AGG TCT TTG CGG ATG TCC ACG T |  |
| HSP90AB1 | CTC TGT CAG AGT ATG TTT CTC GC | GTT TCC GCA CTC GCT CCA CAA A |  |
| CDKN1A | AGG TGG ACC TGG AGA CTC TCA G | TCC TCT TGG AGA AGA TCA GCC G |  |
| PPIA | GGC AAA TGC TGG ACC CAA CAC A | TGC TGG TCT TGC CAT TCC TGG A |  |
| IPO8 | AGG ATC AGA GGA CAG CAC TGC A | AGG TGA AGC CTC CCT GTT GTT C |  |
| PGK1 | CCG CTT TCA TGT GGA GGA AGA AG | CTC TGT GAG CAG TGC CAA AAG C |  |
| ALAS1 | GAT GTC AGC CAC CTC AGA GAA C | CAT CCA CGA AGG TGA TTG CTC C |  |
| PPIH | CAC CTG CTC TAA GTG CGA TTG G | CGA GAT CAC CAC AGG TAG CTT G |  |
| RPL30 | CCA GTC TGT TCT GGC ATG CTT C | CTG GTG TCC ATC ACT ACA GTG G |  |
| RPS18S | GCA GAA TCC ACG CCA GTA CAA G | GCT TGT TGT CCA GAC CAT TGG C |  |
| HMBS | ACG GCT CAG ATA GCA TAC AAG AG | GTT ACG AGC AGT GAT GCC TAC C |  |
| GAPDH | GTC TCC TCT GAC TTC AAC AGC G | ACC ACC CTG TTG CTG TAG CCA A |  |
| HPRT1 | CAT TAT GCT GAG GAT TTG GAA AGG | CTT GAG CAC ACA GAG GGC TAC A |  |
| G6PD | CTG TTC CGT GAG GAC CAG ATC T | TGA AGG TGA GGA TAA CGC AGG C |  |
| NONO | CAT CAA GGA GGC TCG TGA GAA G | TGG TTG TGC AGC TCT TCC ATC C |  |
| TUBB | CTG GAC CGC ATC TCT GTG TAC T | GCC AAA AGG ACC TGA GCG AAC A |  |
| SDHA | GAG ATG TGG TGT CTC GGT CCA T | GCT GTC TCT GAA ATG CCA GGC A |  |
| TBP | TGT ATC CAC AGT GAA TCT TGG TTG | GGT TCG TGG CTC TCT TAT CCT C |  |
| **FOXD1 Targets** | | |  |
| CCNE1 | TGT GTC CTG GAT GTT GAC TGC C | CTC TAT GTC GCA CCA CTG ATA CC |  |
| CDKN1A | AGG TGG ACC TGG AGA CTC TCA G | TCC TCT TGG AGA AGA TCA GCC G |  |
| CDKN1B | ATA AGG AAG CGA CCT GCA ACC G | TTC TTG GGC GTC TGC TCC ACA G |  |
| CDKN2A | CTC GTG CTG ATG CTA CTG AGG A | GGT CGG CGC AGT TGG GCT CC |  |
| FOXP2 | TGG ATG ACC GAA GCA CTG CTC A | TGG GAG ATG GTT TGG GCT CTG A |  |
| PRC1 | ATA GCC AGG AGC AGA GAC AAG C | AAC CGC ACA ATC TCA GCA TCG TG |  |
| WT1 | CGA GAG CGA TAA CCA CAC AAC G | GTC TCA GAT GCC GAC CGT ACA A |  |
| TP53 | CCT CAG CAT CTT ATC CGA GTG G | TGG ATG GTG GTA CAG TCA GAG C |  |
| CTNNB1 | CAC AAG CAG AGT GCT GAA GGT G | GAT TCC TGA GAG TCC AAA GAC AG |  |
| DCN | GCT CTC CTA CAT CCG CAT TGC T | GTC CTT TCA GGC TAG CTG CAT C |  |
| ERK1 | TGG CAA GCA CTA CCT GGA TCA G | GCA GAG ACT GTA GGT AGT TTC GG |  |
| MICU1 | GAC AGT GGC TAA AGT GGA GCT C | CCT CTC ATC AGC CGT TGC TTC A |  |
| PGF | GGC GAT GAG AAT CTG CAC TGT G | ATT CGC AGC GAA CGT GCT GAG A |  |
| PUM1 | GCA TTT GGA CAA GGT CTG GCA G | GCT ACA AGT CGA ACA GGA GCT C |  |
| SLIT2 | CAG AGC TTC AGC AAC ATG ACC C | GAA AGC ACC TTC AGG CAC AAC AG |  |
| **Mitochondrial Metabolism Components** | | |  |
| ANT1 | ATA AGC AGT TCT GGC GCT ACT | GTC CAG CGG GTA GAC AAA GC |  |
| ANT2 | TGA TGG GAT TAA GGG CCT GTA | GAA GTA GGC GGC TCG GTA G |  |
| COXIV | CAG GGT ATT TAG CCT AGT TGG C | GCC GAT CCA TAT AAG CTG GGA |  |
| PDHA1 | TGG TAG CAT CCC GTA ATT TTG | ATT CGG CGT ACA GTC TGC ATC |  |
| PDK1 | GGA TTG CCC ATA TCA CGT CTT T | TCC CGT AAC CCT CTA GGG AAT A |  |
| PDP1 | TGT GAA CTG AGC AGG ATC TAT GG | GGA ATG TAC GAT GAGA GAA CAA CA |  |
| NDUFB8 | ACA GGA ACC GTG TGG ATA CAT | CCC CAC CCA GCA CAT GAA T |  |
| UQRC2 | AAT TTC GTC GTT GGG AAG TAG C | ATG AGT CTG CGG ATT CTG AAA G |  |
| SDHB | ACC TTC CGA AGA TCA TGC AGA | GTG CAA GCT AGA GTG TTG CCT |  |

| Phase of analyzed cells | %Cells* | | %pH3+ | | %yH2AX+ | | %Defects** | |
| --- | --- | --- | --- | --- | --- | --- | --- | --- |
|  | 786-O | FOXD1- | 786-O | FOXD1- | 786-O | FOXD1- | 786-O | FOXD1- |
| **S** | 6.0 | 4.2 | 0 | 0 | 0 | 0 | 0 | 0 |
| **G2** | 21.4 | 12.9 | 100 | 11.1 | 20 | 40 | 0 | 0 |
| **Mitosis** | 60.7 | 62.9 | 60.0 | 0 | 5.3 | 9.1 | 23.5 | 59 |
| Prophase | 21.4 | 14.3 | 50 | 0 | 0 | 33.3 | 11.1 | 30 |
| Metaphase | 16.7 | 32.9 | 71.4 | 0 | 0 | 8.3 | 21.4 | 69.6 |
| Anaphase | 16.7 | 10.0 | 50 | 0 | 16.7 | 0 | 42.9 | 71.4 |
| Cytokinesis | 6.0 | 5.7 | 7.1 | 0 | 0 | 0 | 20 | 25 |
| **Post-mitosis G1** | 7.1 | 18.6 | 0 | 0 | 0 | 83.3 | 16.7 | 100 |
| **Dead/Apoptotic** | 4.7 | 1.4 | - | - | - | - | - | - |


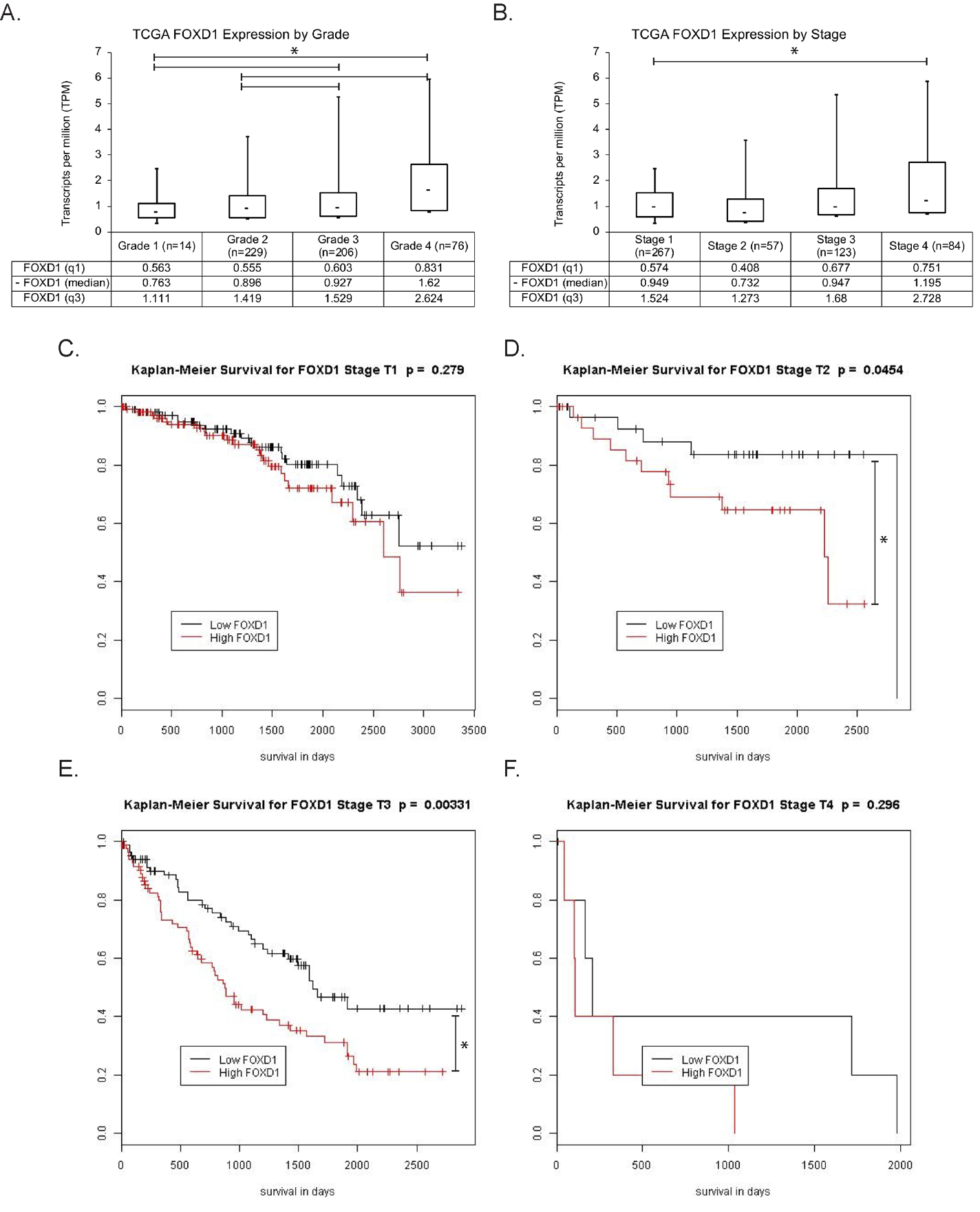


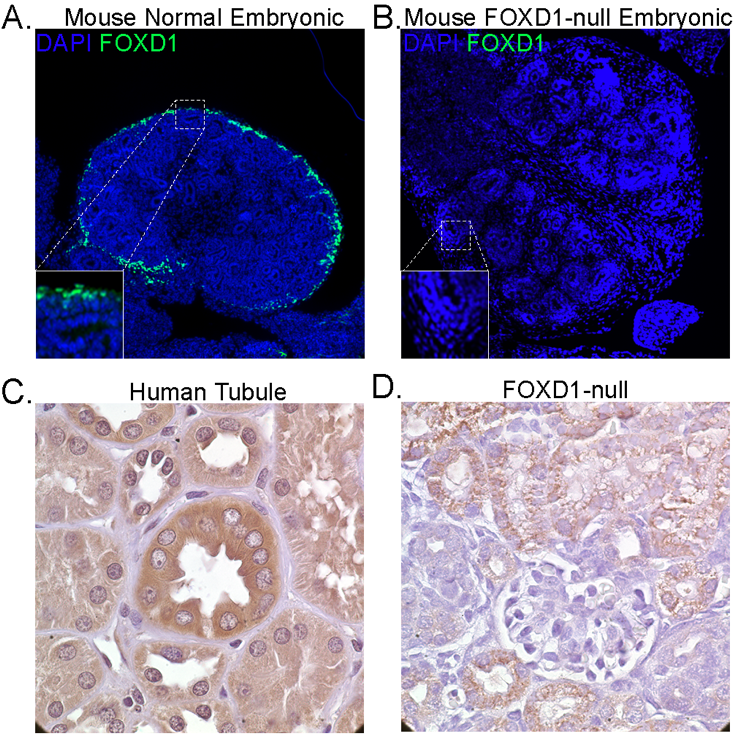


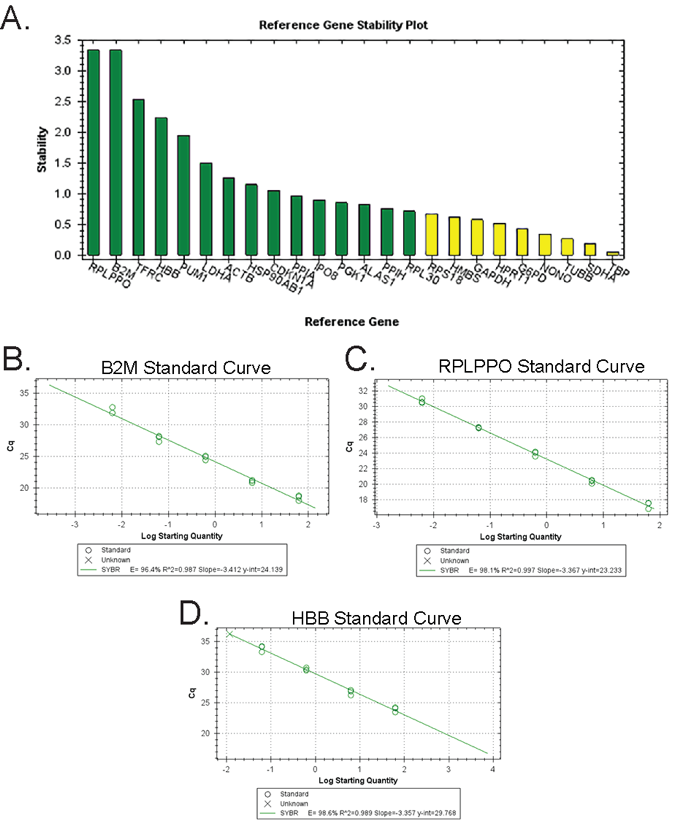


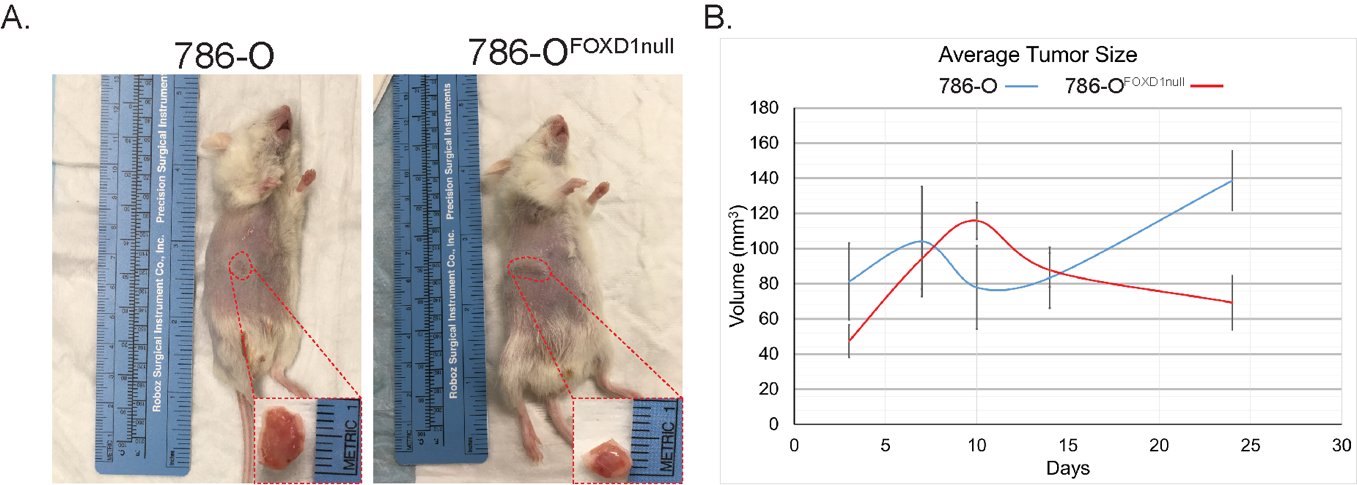


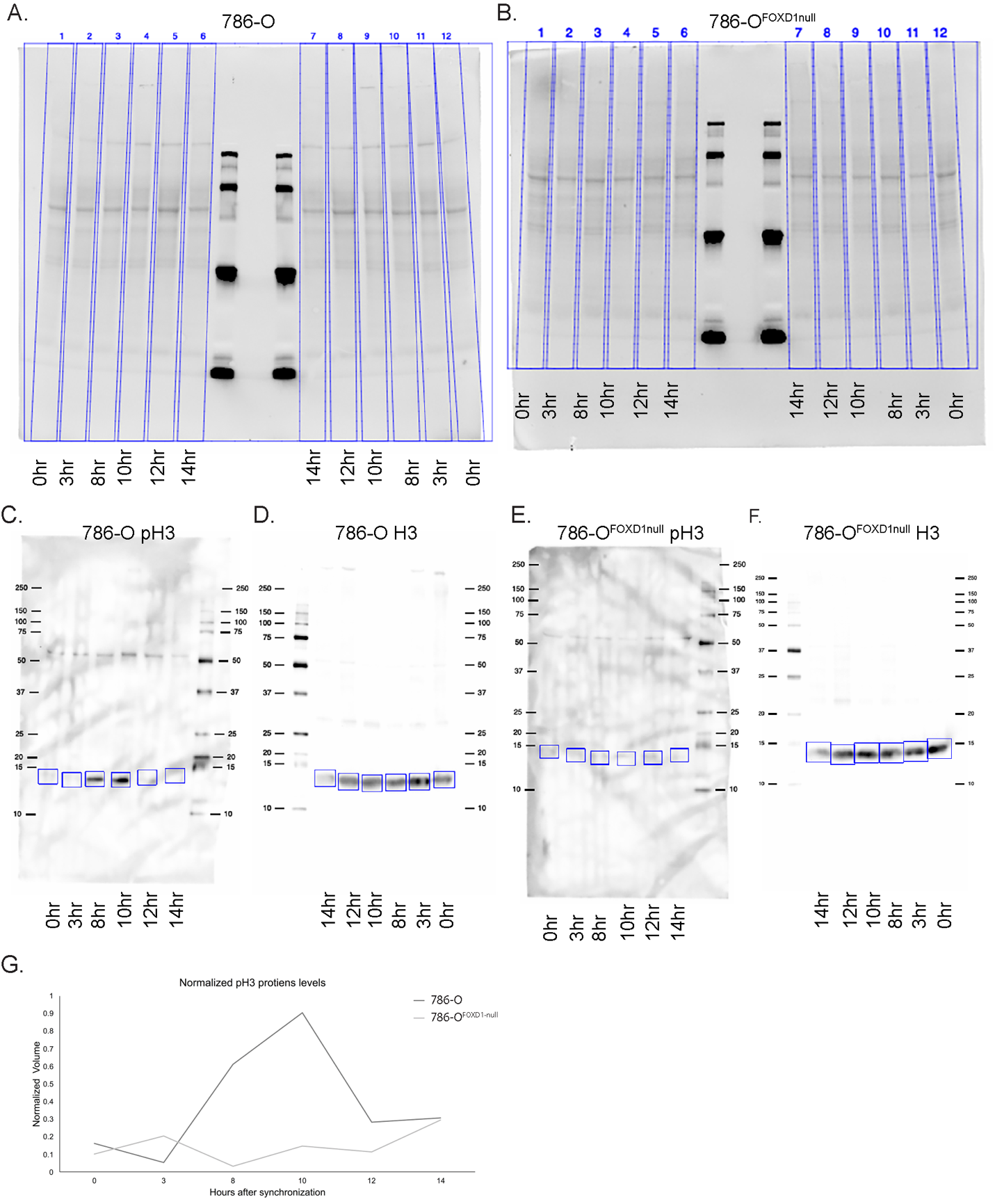


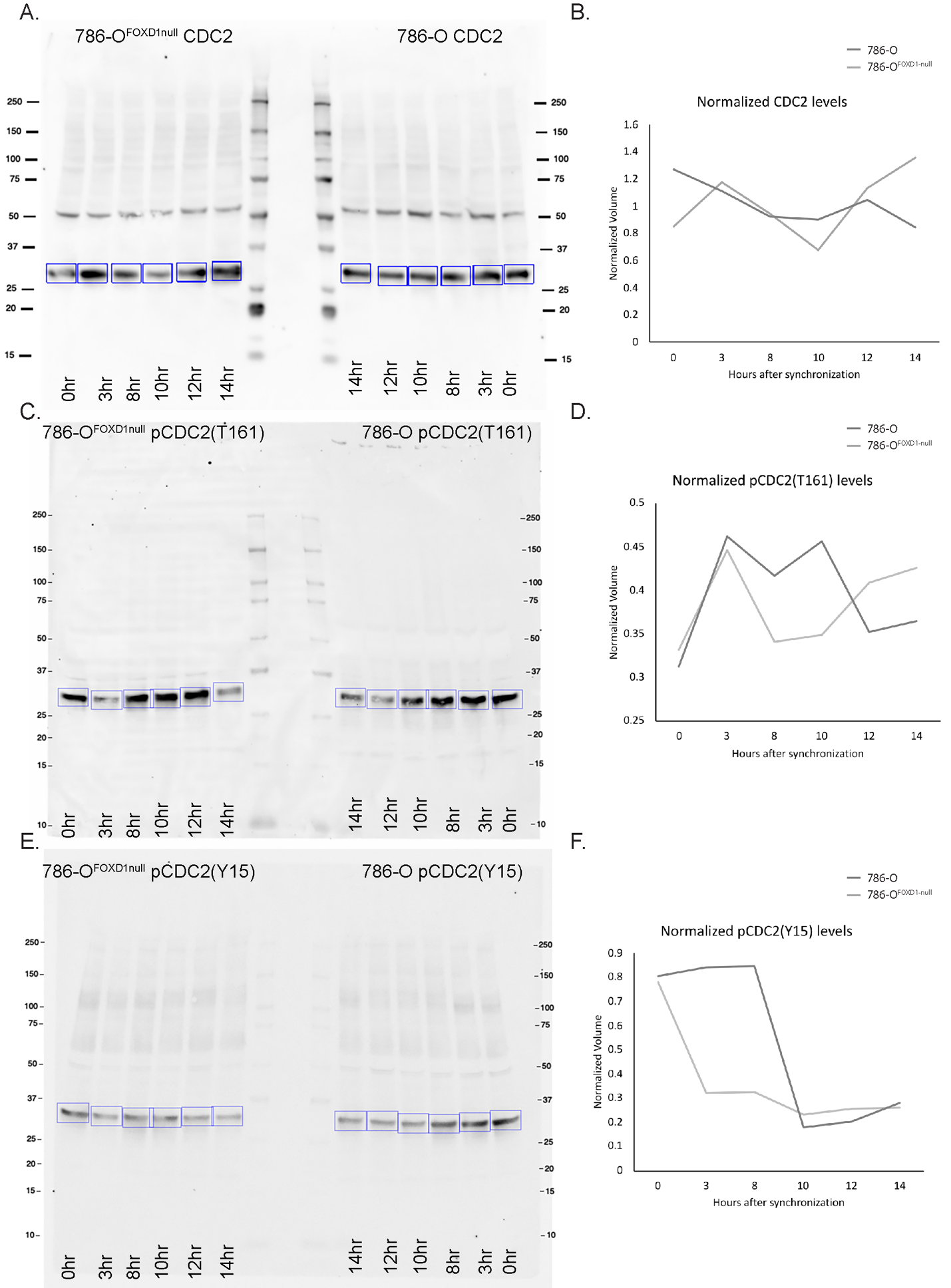


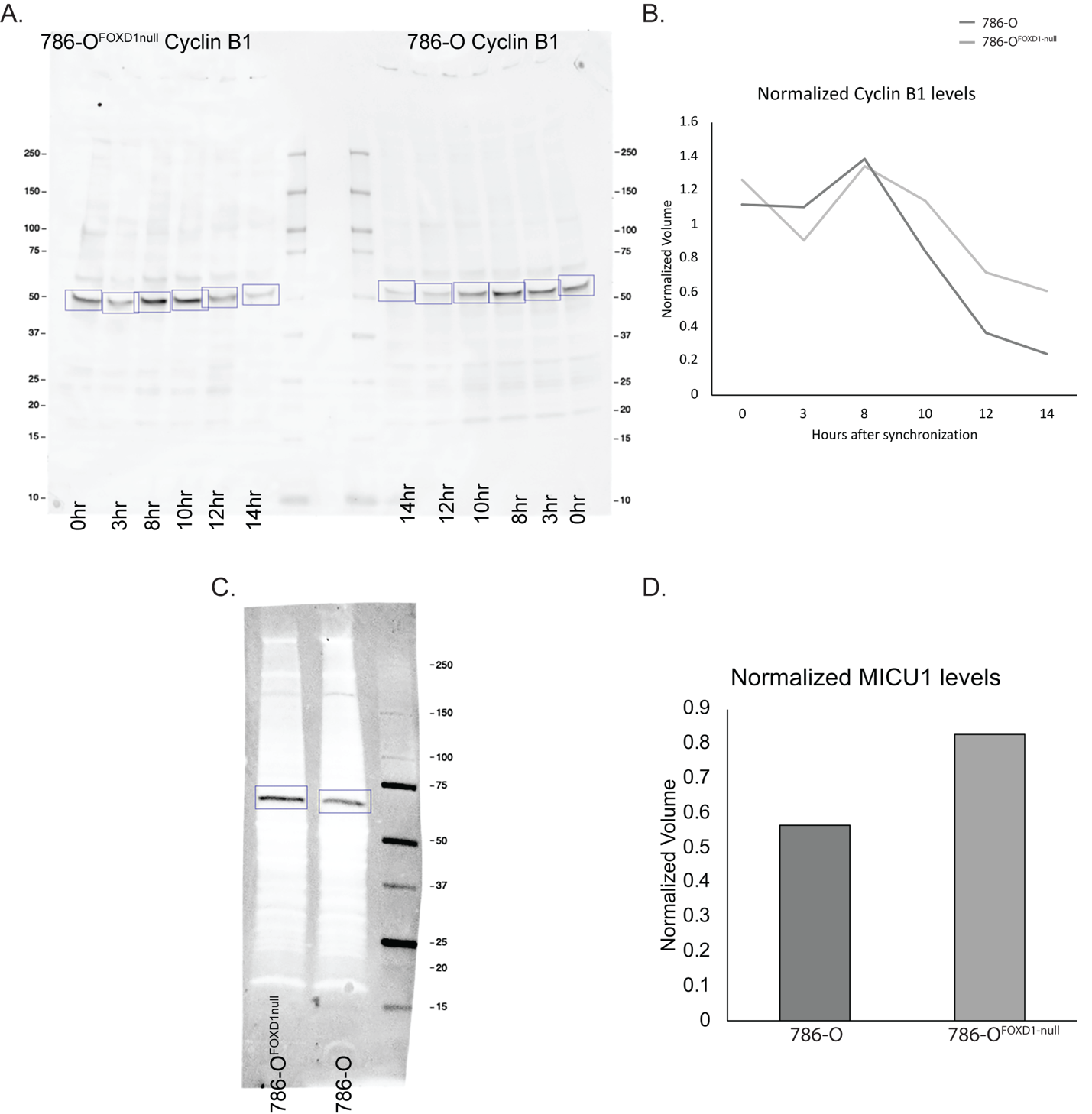


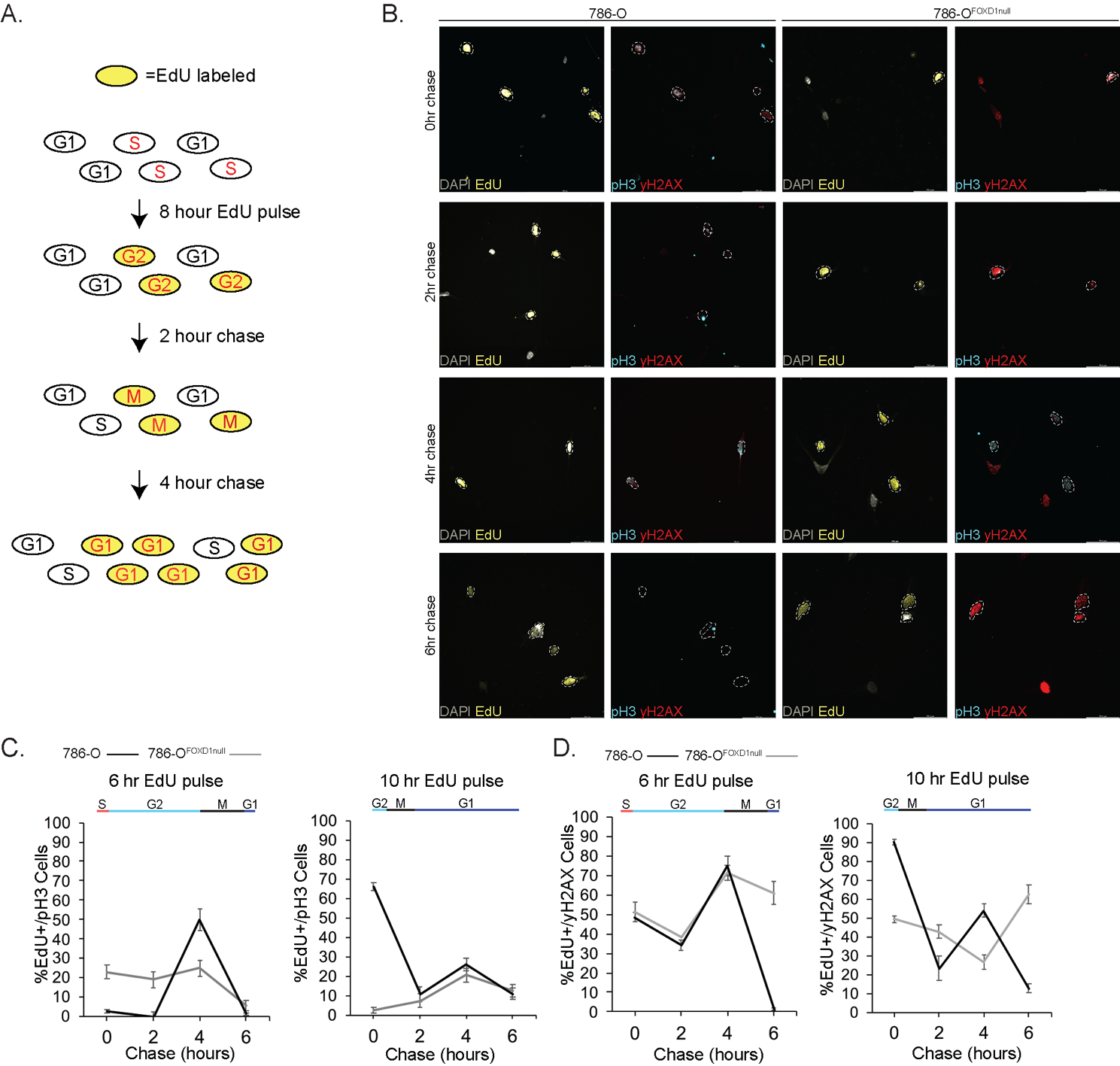


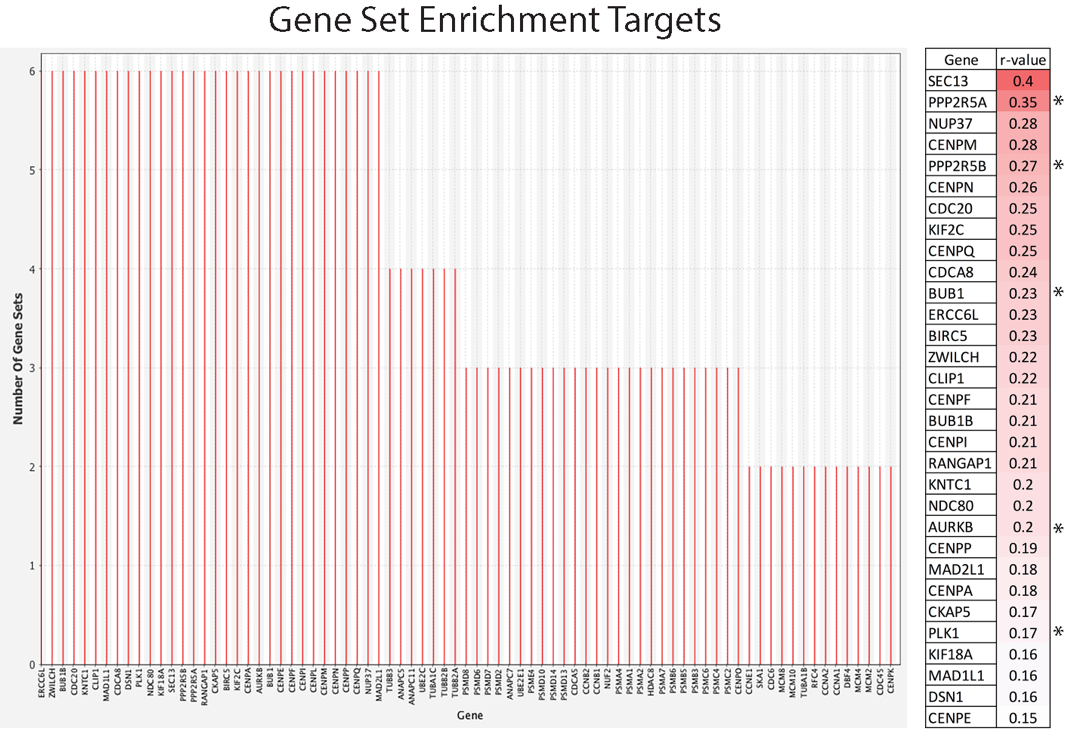

Supplement: Supplementary file 1 — Additional file 1 Table S1: Summary of patient clinical data used for FOXD1 immunohistochemistry analysis. Table S2: Primers used for FOXD1 genotyping and off-target analysis. * Blue indicates primers used for sequencing mismatch region. ** Red bases indicate mismatches from FOXD1. Table S3: Primers for reference genes, FOXD1 targets, and mitochondrial metabolism components. Table S4: Nuclear morphology analysis of 786-O and 786-OFOXD1null. *Immunocytochemistry analysis of cells in different phases of the cell cycle after low density plating, based on nuclear and actin morphologies and localization. Percentage values are based on analysis of 100 cells per group across several fields. **Defects panel indicates collection of possible mitotic defects including lack of chromosome condensation (prophase), loss of spindle polarity (metaphase/anaphase), incomplete sister chromosome separation (cytokinesis), and death of daughter cell (post-mitosis G1). Figure S1: FOXD1 grade and stage analyses. (A) FOXD1 expression level comparisons at different ccRCC tumor grades based on transcriptome data from The Cancer Genome Atlas (B) FOXD1 expression level comparisons at different ccRCC tumor stages based on transcriptome data from The Cancer Genome Atlas. (C-D) Kaplan-Meier survival analyses for ccRCC patients with high versus low tumor expression of FOXD1, analyzed based of tumor stage. *p < 0.05. Figure S2: FOXD1 antibody validation. (A-B) TSA amplification staining for FOXD1 (green) on E12.5 mouse kidneys on normal and FOXD1-null backgrounds. Nuclei counterstained with DAPI (blue), (C-D) Immunohistochemistry staining of adult human kidney tissue (C) and FOXD1-null E12.5 mouse kidney (D). Figure S3: FOXD1 qPCR reference gene selection. (A) Stability scores generated by comparing candidate reference gene assays (Table S3) on equivalent mRNA quantities of 786-O versus 786-OFOXD1null analyzed using the BioRad Reference Gene Selector Tool. Colors of bars denote genes that are over (g [file 12885_2021_7957_MOESM1_ESM.docx]
